# The relationship of symptom dimensions with premorbid adjustment and cognitive characteristics at first episode psychosis: Findings from the EU-GEI study

**DOI:** 10.1016/j.schres.2021.08.008

**Published:** 2021-10

**Authors:** Laura Ferraro, Caterina La Cascia, Daniele La Barbera, Teresa Sanchez-Gutierrez, Giada Tripoli, Fabio Seminerio, Crocettarachele Sartorio, Giovanna Marrazzo, Lucia Sideli, Celso Arango, Manuel Arrojo, Miguel Bernardo, Julio Bobes, Cristina Marta Del-Ben, Charlotte Gayer-Anderson, Hannah E. Jongsma, James B. Kirkbride, Antonio Lasalvia, Sarah Tosato, Pierre-Michel Llorca, Paulo Rossi Menezes, Bart P. Rutten, Jose Luis Santos, Julio Sanjuán, Jean-Paul Selten, Andrei Szöke, Ilaria Tarricone, Roberto Muratori, Andrea Tortelli, Eva Velthorst, Victoria Rodriguez, Andrea Quattrone, Peter B. Jones, Jim Van Os, Evangelos Vassos, Craig Morgan, Lieuwe de Haan, Ulrich Reininghaus, Alastair G. Cardno, Marta Di Forti, Robin M. Murray, Diego Quattrone

**Affiliations:** a Department of Biomedicine, Neuroscience and Advanced Diagnostic (BiND), Section of Psychiatry, University of Palermo, Via G. La Loggia 1, 90129 Palermo, Italy; b Faculty of Health Science, Universidad Internacional de la Rioja (UNIR), Spain; c Department of Child and Adolescent Psychiatry, Hospital General Universitario Gregorio Marañón, School of Medicine, Universidad Complutense, IiSGM (CIBERSAM), C/Doctor Esquerdo 46, 28007 Madrid, Spain; d Department of Psychiatry, Psychiatric Genetic Group, Instituto de Investigación Sanitaria de Santiago de Compostela, Complejo Hospitalario Universitario de Santiago de Compostela, Santiago, Spain; e Barcelona Clinic Schizophrenia Unit, Department of Medicine, Neuroscience Institute, Hospital clinic, University of Barcelona, IDIBAPS, CIBERSAM, Barcelona, Spain; f Department of Medicine, Psychiatry Area, School of Medicine, Universidad de Oviedo, Centro de Investigación Biomédica en Red de Salud Mental (CIBERSAM), C/Julián Clavería s/n, 33006 Oviedo, Spain; g Department of Preventative Medicine, Faculdade de Medicina FMUSP, University of São Paulo, São Paulo, Brazil; h Department of Health Service and Population Research, Institute of Psychiatry, King's College London, De Crespigny Park, Denmark Hill, London SE5 8AF, UK; i Psylife Group, Division of Psychiatry, University College London, 6th Floor, Maple House, 149 Tottenham Court Road, London W1T 7NF, UK; j Section of Psychiatry, Azienda Ospedaliera Universitaria Integrata di Verona, Verona, Italy; k Section of Psychiatry, Department of Neuroscience, Biomedicine and Movement, University of Verona, Piazzale L.A. Scuro 10, 37134 Verona, Italy; l Université Clermont Auvergne, EA 7280, Clermont-Ferrand 63000, France; m Department of Preventive Medicine, Faculdade de Medicina, Universidade of São Paulo, São Paulo, Brazil; n Department of Psychiatry and Neuropsychology, School for Mental Health and Neuroscience, South Limburg Mental Health Research and Teaching Network, Maastricht University Medical Centre, P.O. Box 616, 6200, MD, Maastricht, the Netherlands; o Department of Psychiatry, Servicio de Psiquiatría Hospital "Virgen de la Luz", C/Hermandad de Donantes de Sangre, 16002 Cuenca, Spain; p Department of Psychiatry, School of Medicine, Universidad de Valencia, Centro de Investigación Biomédica en Red de Salud Mental (CIBERSAM), C/Avda. Blasco Ibáñez 15, 46010 Valencia, Spain; q Rivierduinen Institute for Mental Health Care, Sandifortdreef 19, 2333 ZZLeiden, the Netherlands; r INSERM, U955, Equipe 15, 51 Avenue de Maréchal de Lattre de Tassigny, 94010 Créteil, France; s Department of Medical and Surgical Science, Psychiatry Unit, Alma Mater Studiorum Università di Bologna, Viale Pepoli 5, 40126 Bologna, Italy; t Dapertment of Mental Health and pathological addictions, Bologna Local Health Authority, Italy; u Etablissement Public de Santé Maison Blanche, Paris 75020, France; v Department of Psychiatry, Early Psychosis Section, Amsterdam UMC, University of Amsterdam, Meibergdreef 5, 1105 AZ Amsterdam, the Netherlands; w Department of Psychiatry, Icahn School of Medicine at Mount Sinai, New York, USA; x Department of Psychosis Studies, Institute of Psychiatry, Psychology, and Neuroscience, King's College London, De Crespigny Park, Denmark Hill, London SE5 8AF, UK; y National Health Service, Villa Betania Institute, Reggio Calabria, Italy; z Department of Psychiatry, University of Cambridge, Cambridge, UK; aa CAMEO Early Intervention Service, Cambridgeshire & Peterborough NHS Foundation Trust, Cambridge CB21 5EF, UK; ab Department Psychiatry, Brain Centre Rudolf Magnus, Utrecht University Medical Centre, Utrecht, the Netherlands; ac Social, Genetic and Developmental Psychiatry Centre, Institute of Psychiatry, Psychology and Neuroscience, King's College London, London SE5 8AF, UK; ad Department of Psychiatry, Early Psychosis Section, Academic Medical Centre, University of Amsterdam, Amsterdam, the Netherlands; ae Central Institute of Mental Health, Medical Faculty Mannheim, University of Heidelberg, Mannheim, Germany; af Academic Unit of Psychiatry and Behavioural Sciences, University of Leeds, Leeds, UK; ag South London and Maudsley NHS Mental Health Foundation Trust, London, UK

**Keywords:** First episode psychosis, Symptom dimensions, Transdiagnostic, Premorbid adjustment, Cognitive domains, IQ

## Abstract

Premorbid functioning and cognitive measures may reflect gradients of developmental impairment across diagnostic categories in psychosis. In this study, we sought to examine the associations of current cognition and premorbid adjustment with symptom dimensions in a large first episode psychosis (FEP) sample.

We used data from the international EU-GEI study. Bifactor modelling of the Operational Criteria in Studies of Psychotic Illness (OPCRIT) ratings provided general and specific symptom dimension scores. Premorbid Adjustment Scale estimated premorbid social (PSF) and academic adjustment (PAF), and WAIS-brief version measured IQ. A MANCOVA model examined the relationship between symptom dimensions and PSF, PAF, and IQ, having age, sex, country, self-ascribed ethnicity and frequency of cannabis use as confounders.

In 785 patients, better PSF was associated with fewer negative (B = −0.12, 95% C.I. −0.18, −0.06, *p* < 0.001) and depressive (B = −0.09, 95% C.I. −0.15, −0.03, *p* = 0.032), and more manic (B = 0.07, 95% C.I. 0.01, 0.14, *p* = 0.023) symptoms. Patients with a lower IQ presented with slightly more negative and positive, and fewer manic, symptoms. Secondary analysis on IQ subdomains revealed associations between better perceptual reasoning and fewer negative (B = −0.09, 95% C.I. −0.17, −0.01, p = 0.023) and more manic (B = 0.10, 95% C.I. 0.02, 0.18, *p* = 0.014) symptoms. Fewer positive symptoms were associated with better processing speed (B = −0.12, 95% C.I. −0.02, −0.004, *p* = 0.003) and working memory (B = −0.10, 95% C.I. −0.18, −0.01, *p* = 0.024).

These findings suggest that the negative and manic symptom dimensions may serve as clinical proxies of different neurodevelopmental predisposition in psychosis.

## 1 Introduction

Premorbid and cognitive impairment at the first episode of psychosis (FEP) is associated with a worse psychosis outcome (Kravariti et al., 2019; Rabinowitz et al., 2006, Rabinowitz et al., 2005; Díaz-Caneja et al., 2015). However, such impairment is not diagnosis-specific (Arango et al., 2014; Cole et al., 2012; Horton et al., 2015; Larsen et al., 2004; Parellada et al., 2017; Sheffield et al., 2018). Although cognitive impairment in bipolar disorder seems to be generally more severe than in psychotic depression and less severe than in schizophrenia, cognitive function may also vary considerably among patients with the same diagnosis (Sheffield et al., 2018; Trotta et al., 2015; Zanelli et al., 2010).

The transdiagnostic and interindividual variability of premorbid and cognitive factors contributes to the well-known impossibility in setting neat boundaries between the categories of schizophrenia, schizoaffective, and bipolar and major depressive disorders with psychotic features (Laursen et al., 2009; Upthegrove et al., 2017).

Given that premorbid functioning and cognitive measures may reflect differential gradients of developmental impairment in psychosis (Demjaha et al., 2012a; Murray et al., 2004; Owen and O’Donovan, 2017), a model based on continuous symptom dimensions (Quattrone et al., 2019; Van Os et al., 1999) would be methodologically appropriate for examining how these features impact psychosis expression (Guerra et al., 2002; Kravariti et al., 2012; Linscott and van Os, 2010).

Previous studies using symptom dimensions have mostly focused on non-affective psychotic disorders. They showed an association between worse premorbid and cognitive factors with more negative and disorganised, but not positive and depressive symptoms (reviewed by de Gracia Dominguez et al., 2009; Ventura et al., 2010). Interestingly, one study including both non-affective and affective diagnosis at FEP confirmed that negative symptoms were associated with lower IQ and processing speed, while mania showed an inverted-U-shape relationship so that patients with intermediate levels of mania had better cognitive performance than those with low or high levels (Kravariti et al., 2012). Premorbid adjustment in schizophrenia has also been found to be worse in patients with more disorganised (Cohen et al., 2010) and negative symptoms (Bucci et al., 2018b), even when considering FEP samples alone (Chang et al., 2016; Grau et al., 2016; Monte et al., 2008; Stouten et al., 2017); whereas there is mixed evidence on the association between poor premorbid adjustment and positive symptoms (Brill et al., 2009; Bucci et al., 2018b; Chang et al., 2016; Grau et al., 2016; Guerra et al., 2002; MacBeth and Gumley, 2008; Payá et al., 2013; Stouten et al., 2017).

Nevertheless, the paucity of data on premorbid and cognitive features by symptom dimensions in the whole psychosis spectrum (i.e. including both non-affective and affective psychotic disorders) is also caused by the absence of a systematic examination of manic symptoms in the literature (de Gracia Dominguez et al., 2009). Moreover, few studies have been based on large samples of patients at their first episode of psychosis (FEP), which minimise the confounding effect of the course of the disease on cognitive functioning and symptom presentation. In summary, to date, there are no studies that have used the same standardised methodology across multiple countries to evaluate psychopathology at FEP and its relationship with premorbid and cognitive domains.

In the present study, we used a large epidemiological multinational sample to investigate the relationship of both premorbid adjustment and current IQ on the one hand with a bifactor model of psychopathology at FEP on the other. Such a bifactor model comprises 1) a general psychosis factor, which accounts for the variance of all symptoms in the psychosis spectrum; and 2) specific symptom dimensions, that account for the variance due to specific subgroups of symptoms (i.e. positive, negative, disorganisation, manic, and depressive symptoms), independently from the general factor (Reininghaus et al., 2016; Reise et al., 2007). That is, while the general psychosis factor is a measure of the shared symptom presentation in this FEP sample, the specific symptom dimensions instead reflect its heterogeneity.

We expected that premorbid and cognitive measures would account for some heterogeneity in symptom presentation at FEP. Therefore, these measures would explain more variation in specific symptom dimensions than in the general psychosis factor.

More specifically, since the negative symptom dimension can be regarded as a clinical proxy of more neurodevelopmental impairment, we expected that worse premorbid adjustment and cognitive functioning would be associated with more severe negative symptoms. Moreover, we examined if, as opposed to negative symptoms, the manic symptom dimension would indicate less neurodevelopmental impairment, and therefore associated with a better premorbid adjustment and current cognitive functioning.

## 2 Methods

### 2.1 Study design

Subjects were recruited between 01/05/2010 and 01/04/2015 across centres in five different European countries (UK, Italy, Spain, Netherlands, France) and Brazil, as part of *The European Network of National Schizophrenia Networks Studying Gene-Environment Interactions* (EU-GEI) study (http://www.eu-gei.eu/) (CORDIS, 2019; European Network of National Networks studying Gene-Environment Interactions in Schizophrenia (EU-GEI) et al., 2014; Gayer-Anderson et al., 2020; Jongsma et al., 2018; van Os et al., 2008) with the overall aim to examine incidence rates and risk factors of psychotic disorders.

### 2.2 Subjects

Centrally trained researchers screened all potential FEP patients, presenting with a clinical diagnosis for an untreated FEP, even if longstanding, at the mental health services and residents in each catchment area. Since this was a sample of consenting patients derived from an incidence study, inclusion criteria were age 18-64 years and having received operationalised diagnosis of a psychotic disorder (ICD-10: F20-F33) from their clinicians, and confirmed by the researchers, through the Operational Criteria in Studies of Psychotic Illness (OPCRIT) system. Inter-rater reliability was regularly assessed throughout the study. Exclusion criteria were psychotic symptoms precipitated by acute intoxication (ICD10: F1X.5), psychosis due to another medical condition (ICD10: F09) (World Health Organization, 1992), and a previous antipsychotic medication/prescription and/or previous contact with mental health services for psychosis. All local ethical committees approved the study (Gayer-Anderson et al., 2020; Jongsma et al., 2018).

### 2.3 Measures

We collected sociodemographic data using the Medical Research Council (MRC) sociodemographic schedule (Mallett et al., 2002). Psychopathology was rated using the OPCRIT system, a 90-item semi-structured interview, of which 59 items concern psychopathology (McGuffin et al., 1991; Williams et al., 1996). The estimation of general and specific symptom dimensions’ scores was described in full in our previous study (Quattrone et al., 2019) and summarised in the supplementary material. Briefly, the general psychosis factor score measured the covariance due to all psychopathology items, with stronger factor loadings from manic and delusional items. In parallel, the specific symptom dimension scores measured the remainder of variance due to subgroups of positive, negative, disorganisation, manic and depressive items.

IQ was assessed by an abbreviation of the Wechsler Adult Intelligence Scale–III (WAIS-III, ref), including four subtests: Digit Symbol Substitution (Processing Speed), Arithmetic (Working Memory), Block Design (Perceptual Reasoning) and Information (Verbal Comprehension) (Velthorst et al., 2013).

Nine dimensions from the Premorbid Adjustment Scale (PAS) (Cannon-Spoor et al., 1982; Rabinowitz et al., 2007) assessed premorbid social (PSF) and academic (PAF) adjustment in two distinct developmental age-periods: childhood to age 11 and early adolescence (i.e. 12 to age 16) (Rabinowitz et al., 2002; van Mastrigt and Addington, 2002). PSF included social withdrawal (<11 and 12-16 years), peer relationships (<11 and 12-16 years), and ability to form socio-sexual relationships (designed from 12 to age 16 only). PAF included scholastic performance (<11 and 12-16 years), and adaptation to school (<11 and 12-16 years) (Ferraro et al., 2019). IQ calculation and the PAS reduction of dimensions (Ferraro et al., 2019) included in these analyses were described in our previous studies, also summarised in the supplementary material.

Date of onset and weeks of untreated psychosis (DUP) were recorder by the Nottingham Onset Scale (Singh et al., 2005). Thus, for retrospective interviews, we ensured that they referred to the pre-onset period (Gayer-Anderson et al., 2020). A medication list was recorded, including antipsychotic (AP) treatment, codified as AP free/ 1 AP /more than 1 AP. The Cannabis Experience Questionnaire (CEQ_EU-GEI_) evaluated the frequency of cannabis use (Di Forti et al., 2019).

The core assessment (Gayer-Anderson et al., 2020) was administered as soon as the patient was compliant; psychopathology was rated by clinicians referring to any symptoms experienced within the first month of FEP; the WAIS and other retrospective measures (such as PAS) were proposed when the patient mental state was stable, as confirmed by his/her clinicians.

### 2.4 Statistical analysis

The analysis was performed following two steps in SPSS 25 (IBM Corporation, 2017).

First, we used multivariable analysis of the covariance (MANCOVA) to test the difference in symptom dimensions (the output), based on concomitant discrete and continuous independent variables (age, sex, self-reported ethnicity [white, black, or other ethnicities], study country, PSF, PAF, and IQ scores), so that the relationships found between one dimension and the predictors accounted (a) for those between the others, (b) and the intra-individual correlation of the dimensions in their severity. Given our previous reports in the EU-GEI study of an association between frequency of lifetime cannabis use with more positive and fewer negative symptoms (Quattrone et al., 2020), as well as better PSF and IQ (Ferraro et al., 2019, Ferraro et al., 2013), we added this variable as an additional ordinal predictor (i.e. never use = 0; occasional/less than daily use = 1; daily use = 2) (Ferraro et al., 2019). Box's M test examined the covariance matrix, and effect size estimates were evaluated by Pillai's trace and partial eta squared.

Second, we aimed to investigate if any specific cognitive function had a stronger relationship with the outcome than others. Thus, exploratory post hoc analyses were run by follow-up ANCOVAs with IQ subtests, PSF, PAF as the independent predictors, and age, sex, self-reported ethnicity, and study country as confounders, and those symptom dimensions where IQ had an effect as the dependent variables (inserted once at a time). Moreover, we were interested to test frequency of cannabis use role in those specific symptom dimensions where it had an effect, and its interactions with premorbid adjustment and current IQ scores if appropriate (i.e., if both terms were predictive per se). All comparisons in MANCOVA and ANCOVAs were Bonferroni-adjusted and further corrected using the Benjamini-Hochberg (B—H) procedure, pre-determining a 5% chance of a false discovery rate.

Following-up the Kravariti et al. (2012) study, we used a post hoc curve estimation regression to explore whether the association between manic symptoms and cognitive performance followed a U-shape (quadratic) better than a linear relationship, based on Akaike and Bayesian Information Criteria (AIC and BIC).

Finally, we wanted to repeat the MANCOVA by including DUP and AP treatment as covariates, to avoid any influence of long-lasting symptomatology and pharmacotherapy on the relationship between the variables of interest.

## 3 Results

### 3.1 Descriptive characteristics

785 out of 1130 FEP patients from the original sample (Di Forti et al., 2019; Gayer-Anderson et al., 2020) had a complete set of information for symptom dimensions, IQ, PAF and PSF, and confounding and mediating variables. The subjects included in this sample were not different from the rest of the sample in terms of sex (61.3% males vs. 62.6% males, χ^2^(1) = 0.18, *p* = 0.67) and ethnicity (65.2% white vs. 58.8% white, χ^2^(2) = 4.73, *p* = 0.09), but they were younger (Mean 30.5 (sd = 10.3) vs. Mean 33 (sd = 11.1)), t(2,1128) = 3.7, *p* < 0.001). This sample had a lower proportion of patients from the UK (18.5%vs. 29.3% vs.) and Italy (11.8% vs. 27.2), as compared to the rest of the sample (χ^2^(5) = 94.8, p < 0.001).

The mean age of the sample was 30.5 years (sd = 10.3), and the majority of the subjects were male (61.3% [*N* = 481]), white (65.2% [*N* = 512]), unemployed (56.7% [*N* = 445]), single (64.5% [*N* = 506]), and living with their parents or another family (58.1% [*N* = 456]) at the time of the interview. 42.7% (*N* = 335), had a first-level (professional, A-level) education. Supplementary Table 1 mean IQ of the study sample was 85.5 (sd = 18.1), and 66% (*N* = 518) of the subjects had used cannabis in their lifetime (Table 1). 75.5% (*N* = 593) of the patients received a diagnosis of “non-affective psychosis” (32.3%, *N* = 253 schizophrenia) as detailed in Supplementary Table 1. Supplementary Table 2 describes the crude correlations between symptom dimensions and premorbid adjustment and current cognitive functioning.

**Table 1:** Descriptive characteristics of the sample.

Country, N (%)		
UK	145	(18.5)
Netherlands	167	(21.3)
Spain	168	(21.4)
France	64	(8.2)
Italy	93	(11.8)
Brazil	148	(18.9)
Age, Mean (sd)	30.5	(10.3)
Gender, N (%)		
Males	481	(61.3)
Females	304	(38.7)
Self-reported ethnicity, N (%)		
White	512	(65.2)
Black	117	(14.9)
Other	156	(19.9)
Occupation, N (%)		
Employed, student	327	(41.7)
Unemployed, economically inactive	445	(56.7)
Education, N (%)		
University and post-graduated	130	(16.6)
First-level (job related, A-level)	335	(42.7)
Compulsory education	206	(26.2)
No education	110	(14.0)
Relationship Status, N (%)		
Married, in a steady relationship	229	(29.2)
Divorced, separated, widowed	48	(6.1)
Single	506	(64.5)
Living Status, N (%)		
Partner, with children, friends	210	(26.8)
Alone	112	(14.3)
Parents, other family, other	456	(58.1)
Lifetime frequency of cannabis use, N (%)		
Never	267	(34.0)
Occasionally	274	(34.9)
Everyday	244	(31.1)
IQ, Mean (sd)	85.5	(18.1)

### 3.2 Predictive model for symptom dimensions by premorbid adjustment and cognition

The MANCOVA analysis showed that IQ [Pillai = 0.019, F(6, 765) = 2.52; *p* = 0.020; partial η^2^ = 0.019] and premorbid social impairment (PSF) [Pillai = 0.032, F(6, 765) = 4.26; *p* < 0.001; partial η^2^ = 0.032] had an overall discriminant effect of a small magnitude on symptom dimensions, whereas academic premorbid impairment (PAF) did not [Pillai = 0.007, F(6, 765) = 0.93; *p* = 0.468; partial η^2^ = 0.007] (Table 2).

**Table 2:** Multivariate tests discriminant effects^a^.

Effect	Pillai's Trace	F	df	Error df	*p-Value*	Partial η^2^
Intercept	0.04	5.253	6	765	<0.001	0.04
Country	0.321	8.8	30	3845	**<0.001**	0.064
Gender	0.019	2.49	6	765	**0.022**	0.019
Ethnicity	0.029	1.872	12	1532	**0.034**	0.014
Age	0.051	6.912	6	765	**<0.001**	0.051
PSF	0.032	4.266	6	765	**<0.001**	0.032
PAF	0.007	0.937	6	765	0.468	0.007
IQ	0.019	2.527	6	765	**0.02**	0.019
Frequency of cannabis use	0.027	1.763	12	1532	**0.049**	0.014

a Design: Intercept + country + gender + ethnicity + age + PSF + PAF + IQ + frequency of cannabis use.

Positive symptoms were slightly more common in individuals with lower IQ (B = -0.005, 95% C.I. -0.01, 0.0, *p* = 0.033). Negative symptoms were associated with lower IQ, although the magnitude of the association was very small (B = -0.005, 95% C.I. -0.01, -0.001, *p* = 0.014), and with worse PSF (B = -0.12, 95% C.I. -0.18, -0.06, p < 0.001). By contrast, manic symptoms were slightly more common in patients with higher IQ (B = 0.005, 95% C.I. 0.0004, 0.009, p = 0.03), and better PSF (B = 0.07, 95% C.I. 0.14, 0.01, *p* = 0.023). Depressive symptoms were more common in individuals with lower PSF (B = -0.09, 95% C.I. -0.15, -0.03, *p* = 0.032). Finally, we did not observe an association between IQ and/or PSF with the general psychosis factor or the disorganisation symptom dimensions (Table 3).

**Table 3:** Parameter estimates in the predictive model for symptom dimensions by PSF, PAF and IQ.

Dependent variable	Parameter^a^	B	SE	t	*p-Value*	95% C.I.		Partial Eta^2^
						Lower Bound	Upper Bound	
GENERAL	Intercept	0.049	0.205	0.24	0.81	−0.353	0.451	0.000
	PSF	0.047	0.027	1.707	0.088	−0.007	0.101	0.004
	PAF	0.042	0.031	1.339	0.181	−0.019	0.103	0.002
	IQ	−0.001	0.002	−0.536	0.592	−0.005	0.003	0.000
	Daily vs never use	−0.1	0.082	−1.216	0.224	−0.262	0.061	0.002
	Daily vs occasional use	−0.078	0.073	−1.068	0.286	−0.222	0.066	0.001
POSITIVE	Intercept	0.313	0.264	1.187	0.236	−0.205	0.832	0.002
	PSF	−0.041	0.035	−1.155	0.248	−0.11	0.029	0.002
	PAF	−0.04	0.04	−0.984	0.325	−0.119	0.039	0.001
	IQ	−0.005	0.002	−2.133	**0.033**	−0.01	0.000	0.006
	Daily vs never use	−0.27	0.106	−2.539	**0.011**	−0.478	−0.061	0.008
	Daily vs occasional use	−0.256	0.095	−2.705	**0.007**	−0.442	−0.07	0.009
NEGATIVE	Intercept	0.383	0.243	1.577	0.115	−0.094	0.859	0.003
	PSF	−0.124	0.033	−3.821	**0.000**	−0.188	−0.06	0.019
	PAF	−0.033	0.037	−0.89	0.374	−0.105	0.04	0.001
	IQ	−0.005	0.002	−2.452	**0.014**	−0.01	−0.001	0.008
	Daily vs never use	0.19	0,098	1.946	0.052	−0.002	0.381	0,005
	Daily vs occasional use	0.018	0,087	0.202	0.84	−0.153	0.188	0
DISORGANIZATION	Intercept	0.458	0.238	1.924	0.055	−0.009	0.926	0.005
	PSF	−0.056	0.032	−1.746	0.081	−0.118	0.007	0.004
	PAF	0.041	0.036	1.132	0.258	−0.03	0.112	0.002
	IQ	−0.003	0.002	−1.497	0.135	−0.008	0.001	0.003
	Daily vs never use	−0.09	0.096	−0.936	0.349	−0.278	0.098	0.001
	Daily vs occasional use	−0.072	0.085	−0.843	0.4	−0.239	0.096	0.001
MANIA	Intercept	0.365	0.246	1.485	0.138	−0.118	0.848	0.003
	PSF	0.075	0.033	2.275	**0.023**	0.01	0.14	0.007
	PAF	−0.014	0.037	−0.374	0.709	−0.088	0.06	0.000
	IQ	0.005	0.002	2.172	**0.03**	0.000	0.009	0.006
	Daily vs never use	−0.161	0.099	−1.628	0.104	−0.355	0.033	0.003
	Daily vs occasional use	0.038	0,088	0.436	0.663	−0.135	0.212	0
DEPRESSION	Intercept	−0.244	0.236	−1.033	0.302	−0.708	0.22	0.001
	PSF	−0.093	0.032	−2.941	**0.003**	−0.155	−0.031	0.011
	PAF	0.013	0.036	0.37	0.712	−0.057	0.084	0.000
	IQ	−0.002	0.002	−1.122	0.262	−0.007	0.002	0.002
	Daily vs never use	0.058	0,095	0.612	0.541	−0.128	0.245	0
	Daily vs occasional use	0.065	0,085	0.765	0.444	−0.101	0.231	0.001

Gender, Age, ethnicity and Country parameters are showed in the Supplementary Table 4.

A small effect on the model was observed for cannabis use on symptom dimensions [Pillai = 0.027, F(12, 1532) = 1.76; *p* = 0.049; partial η^2^ = 0.014] (Table 1). Specifically, positive symptoms were higher in individuals with daily use of cannabis, compared with never (B = −0.270, 95% C.I. −0.478, −0.061, *p* = 0.011) and occasional users (B = −0.256, 95% C.I. −0.442, −0.07, *p* = 0.007). Other variables’ effects are described in the supplementary material.

A post hoc exploratory analysis was performed removing IQ from the predictors to examine whether the lack of association between PAF and the scores on any of the symptom dimensions was due to the current cognitive measures in the model, but this was not the case [Pillai = 0.012, (6, 766) = 1.5; *p* = 0.168; partial η^2^ = 0.012].

A longer DUP was associated with more positive symptoms (*r* = 0.170, *p* < 0.001) and with lower scores at the general symptom dimension (r = -0.124, *p* = 0.001). Moreover, a longer DUP correlated with lower PSF (r = -0.124, p = 0.001), but not with differences in IQ (*r* = 0.005, *p* = 0.901). However, the results described above did not change when adjusted by the DUP and the number of AP taken at the interview time (see supplementary material).

### 3.3 The specific effect of IQ subtests on symptom dimensions

We then examined the effect of processing speed, working memory, perceptual reasoning, and verbal comprehension on negative, manic and positive dimensions, with the additional confounder effect of frequency of cannabis use on the positive symptom dimension only.

Associations of small magnitude were observed for perceptual reasoning with a lower score on the negative symptom dimension (B = −0.09, 95% C.I. −0.17, −0.01, *p* = 0.023) and a higher score on the manic symptom dimension (B = 0.10, 95% C.I. 0.02, 0.18, *p* = 0.014).

In a post hoc analysis, we found that the quadratic term (AIC = 2266.346; BIC = 2317.972) did not improve the model compared with the linear one (AIC = 2265.142; BIC = 2316.768). Thus, we maintained the assumption of a linear relationship between manic symptoms and perceptual reasoning.

Associations of small magnitude were also observed for worse processing speed (B = −0.12, 95% C.I. −0.02, 0.004, *p* = 0.003) and working memory (B = −0.10, 95% C.I. −0.18, −0.01, *p* = 0.024) and a higher score on the positive symptom dimension.

Higher frequency of cannabis use was moderately associated with higher positive symptom scores (never vs daily use = B = 0.25, 95% CI 0.04, 0.46, *p* = 0.016; occasional vs daily use = B = 0.24, 95% CI 0.05, 0.42, *p* = 0.010).

We found evidence for a positive symptom-by-cannabis use interaction effect, suggesting that processing speed is differently associated with positive symptoms, depending on frequency of cannabis-use (F(2, 767) = 3.1, *p* = 0.047, partial η^2^ = 0.008). Specifically, cannabis daily-users had more positive symptoms than never- (B = 0.273, 95% C.I. 0.02, 0.52, *p* = 0.030) and occasional users (B = 0.245, 95% C.I. 0.019, 0.47, *p* = 0.029), regardless of their processing speed scores. However, within the group of occasional cannabis users, positive symptoms were fewer when processing speed deficits were less pronounced (B = −0.22, 95% C.I. −0.4, −0.03, *p* = 0.017, partial η^2^ = 0.007) (Supplementary-Fig. 1).

## 4 Discussion

First, we found that premorbid social functioning was independently associated with specific patterns of symptom presentation. FEP patients with lower levels of social adjustment presented with more negative and depressive symptoms, whereas those with higher levels of social adjustment presented with more manic symptoms.

Second, we found an independent, small association between IQ and symptom dimensions. Specifically, patients with current lower IQ presented with slightly more negative and positive symptoms and fewer manic symptoms compared with those with higher IQ scores. However, these associations were of small magnitude.

Third, when considering IQ sub-tests, we found that specific cognitive sub-domains drove the association between IQ and symptom presentation. That is, while lower processing speed and working memory were associated with more positive symptoms, lower perceptual reasoning was associated with more negative and fewer manic symptoms.

### 4.1 Manic and negative symptoms

Our findings are congruent with previous studies focused on individuals at risk for psychosis (Tarbox et al., 2013) and around the onset of first psychosis. (Payá et al., 2013; Chang et al., 2013; Horton et al., 2015), suggesting that variation in cognitive and premorbid social features is associated with different early trajectories of premorbid adjustment.

In our study, higher IQ and better premorbid sociability were associated with more manic symptoms and fewer negative symptoms, which is in line with the suggested heterogeneity in neurodevelopmental predisposition in psychosis. Indeed, negative symptoms at FEP are often considered a marker of early developmental impairment (Murray and Castle, 1991).

When considering diagnoses, more severe neurodevelopmental impairment has been extensively reported in schizophrenia than in bipolar disorders (Allardyce et al., 2007; Bucci et al., 2018a; Koenen et al., 2009; Mollon et al., 2018; Torrent et al., 2018; Trotta et al., 2015). Altogether, these elements support the hypothesis of a continuous distribution of neurodevelopmental impairment across the psychosis spectrum and within disorders; to some extent, the categories of schizophrenia and bipolar disorder might approximately catch the high and low extremes of this distribution (Arango et al., 2014; Demjaha et al., 2012b; Murray et al., 2004; Parellada et al., 2017).

The association between IQ and negative and manic dimensions was largely driven by the perceptual-reasoning domain, which is related to perceptual abnormalities (Morita et al., 2019; Silverstein and Keane, 2011) and to the speed of thinking process (Scheiber et al., 2017), which is accelerated in manic episodes and decelerated in negative states. Perceptual-reasoning is also considered a proxy of ‘general ability index’ of intelligence, able to overcome subjects’ linguistic problems (Green et al., 2008), and it is generally more impaired in schizophrenia compared with other psychotic disorders.

We reported this association using a delimited negative symptom dimension composed of restrictive and blunted affect and poverty of speech.

These findings are in line with one previous report on FEP patients concerning negative and manic symptoms (Kravariti et al., 2012). However, unlike Kravariti et al. (2012), we did not find an inverse U-shape relationship between cognition and manic symptoms. Of note, we choose to do not exclude patients with IQ < 70; this contributed to lower the median IQ of the sample, but it made our findings reflecting the real clinical practice.

### 4.2 Positive symptoms

Positive symptoms were associated with lower current IQ but not with worse premorbid adjustment. This relationship was driven by lower processing speed and worse working memory, which are well-recognised early neuropsychological markers of schizophrenia (Dickinson et al., 2007; Forbes et al., 2009; Lee and Park, 2005; Silver et al., 2003). On the other hand, a deficit in working memory has been reported both in individuals with schizophrenia and bipolar disorder currently experiencing auditory verbal hallucinations (Jenkins et al., 2018) that may be linked to dysfunctions in verbal memory encoding (Gisselgård et al., 2014).

Processing speed may relate to a broad range of cognitive dysfunctions, more than merely low-speed (Ayesa-Arriola et al., 2016; Rodríguez-Sánchez et al., 2007), and it implies encoding abilities as well (Mathias et al., 2017).

Indeed, these cognitive sub-domains are probably associated to fluid intelligence (Fry and Hale, 2000; Neisser et al., 1996; Yuan et al., 2006), and their functioning increases in concert as the effect of the cognitive-developmental cascade (Fry and Hale, 2000). Thus, their dysfunction might precede or be contextual to the diagnosis and can contribute to the aberrant coding typical of positive symptoms (Freeman et al., 2014; Laloyaux et al., 2018; Zhu et al., 2018). Given the result of our supplementary analysis, including the effect of AP medication, it is unlikely that these results are related to the use of antipsychotics at the time of the interview.

Of note, fluid intelligence is not only state-, but also and trait-dependent. Hence, we cannot firmly establish a causal direction of this association between positive symptoms and lower processing speed and general IQ. However, more insight on it can derive from the interactive effects of patterns of cannabis use.

The frequency of cannabis use was associated with more prominent positive symptoms at FEP (Ringen et al., 2016; Seddon et al., 2016), in a dose-response effect (Quattrone et al., 2020). Particularly, we found an interaction between cannabis use and processing speed in predicting positive symptoms in the group of occasional cannabis users. Such a group of patients may present with the most cognitively-related positive symptoms, i.e. being not related to neurodevelopmental impairment, compared with never users (Ferraro et al., 2019), as well as to long-lasting or residual effects of cannabis use, compared with daily users (Quattrone et al., 2020) (see supplementary material). Therefore, it is intriguing to speculate whether cannabis use could be considered as a transdiagnostic proxy of less neurodevelopmental impairment in psychotic disorders. Notably, never users presented with the lowest positive symptoms, regardless of their processing speed abilities, in line with the finding of a predominance of negative symptoms in this subgroup (Pope et al., 2021; Quattrone et al., 2020).

### 4.3 Depressive symptoms

Worse premorbid sociability but not current cognition was associated with depressive symptoms, in line with previous studies (Tarbox et al., 2012). These findings support a difference between mood downregulation and flat affect, the second being more a cognitively-related deficit in the emotion-processing, while depressive symptom dimension in psychosis could be part of a motivational impairment, independent from negative symptoms (Culbreth et al., 2018), which needs further attention from both a research and clinical point of view (Upthegrove et al., 2017).

### 4.4 Disorganised symptoms

Disorganisation is often indicative of a lower psychosocial functioning (Minor et al., 2015; Minor and Lysaker, 2014); however, in our sample, we did not find any relationship of current cognitive characteristics and premorbid adjustment with disorganisation symptoms at FEP. Consistent with previous literature (Díaz-Caneja et al., 2015; Drake et al., 2016; Galderisi et al., 2012; Morgan et al., 2008; Petruzzelli et al., 2018), men in our sample showed more disorganised and negative symptoms than women; furthermore, an earlier age-of-onset was related to more disorganised and negative and fewer depressive symptoms, in line with hypothesised sex-related neurodevelopmental characteristics of psychosis (Murray and Castle, 1991; Riecher-Rössler and Häfner, 2000; Seeman, 1997).

### 4.5 General psychosis factor

Finally, we found that the general psychosis factor was not associated with cognitive and premorbid features. This firstly suggests that current cognitive characteristics and premorbid adjustment at FEP may reflect specific symptom-developmental predisposition as opposed to unspecific, common psychopathology. Moreover, this finding should be put in the context of the general factor conceptualisation in the EU-GEI FEP sample (Quattrone et al., 2019), where the identified general psychosis factor catch the shared characteristics across diagnostic categories of non-affective and affective psychosis (Quattrone et al., 2019). Of note, the conceptualisation of a general factor in our sample, and its relationship with cognitive and premorbid features, may be different from samples that include all psychiatric diagnoses (Kotov et al., 2020), or the general population (Caspi et al., 2020; Murphy et al., 2020). In such scenarios, the general factor reflects a wider range of symptoms than psychosis, and it may therefore serve as a more general index of psychopathology.

## 5 Limitations

This study has two main limitations. First, we used a narrow negative symptom dimension due to the limited coverage of negative symptoms of the OPCRIT (Quattrone et al., 2019). Still, our findings are in line with previous literature, and they are, overall, coherent with a developmental risk model of psychosis (Murray et al., 2017). Second, we cannot exclude some extent of recall bias in referring to premorbid adjustment using the PAS. However, the PAS is the most validated tool for measuring premorbid adjustment in patients who have been diagnosed with a psychotic disorder (Brill et al., 2008; Rabinowitz et al., 2007). Finally, our PAS measures stop at age 16 (early adolescence), to prevent overlap with illness onset, while previous studies reported the most significant deterioration between 16 and 18 years (late adolescence) for the academic factor (PAF) (Allen et al., 2013; Strauss et al., 2012). This could be responsible for the counterintuitive finding of a lack of association between a worse PAF and symptomatological markers of neurodevelopmental impairment, such as negative or depressive symptoms. On the other hand, one of these studies (Strauss et al., 2012) reported a greater premorbid PAF impairment in those with less PSF deficiency, thus suggesting a non-linear association between these two factors. Third, this is a multinational study and, as expected, we observed differences in symptom dimension scores across countries. We consider these differences as reflecting the genuine variation of psychopathology in different contexts. Indeed, all researchers underwent a psychopathology OPCRIT rating and inter-rater reliability was calculated before and during the study. Moreover, country, used as a confounder, did not affect our main findings, which are in turn more generalizable due to the multinational design of the EU-GEI study.

## 6 Conclusions

Our findings support the use of symptom dimensions in psychosis research and clinical practice. Indeed, the plausibility of such continuous phenotypes depends on both their validity and the degree of useful information that can be derived by their examination.

From a research perspective, we evaluated a dimensional symptom-developmental model of psychosis. In our sample, cognitive abilities and premorbid functioning contributed to the heterogeneous expression of psychosis at FEP. Assuming that dysfunction in current cognitive and premorbid social abilities are indicative of more neurodevelopment impairment in psychosis, we may speculate that our findings reflect the existence of different neurodevelopmental predispositions in psychosis, resulting in differential symptomatology at FEP.

From a clinical perspective, the identification of premorbid and cognitive features associated with symptom presentation may be relevant to overcome certain issues with psychiatric nosology. In addition to the traditional diagnoses, mental health professionals should be encouraged to formulate enhanced clinical impressions, integrating their evaluation of symptoms at FEP with information on personal history and cognitive functioning. Our findings suggest that premorbid academic poor adjustment could be a less sensitive risk-factor than an early social poor adjustment, which would require further attention and consideration in future studies, where the use of both these measured would be highly recommended.

## CRediT authorship contribution statement

Authors LF and DQ designed the study and wrote the protocol, they made statistical analyses and wrote the first draft of the manuscript. Authors CLC, TSG, GT, FS, CS, GM and LS helped with the literature searches and the revision of the manuscript.

Authors DLB, CA, MB, EV, PBJ, CM, HEJ, JBK, ST, PML, JPS, IT, RM, JVO, EV, LdH, AGC, MDF provided valuable feedback on previous versions and approved the final manuscript. RMM provided his valuable feedback, supervised on previous versions and approved the final manuscript.

## Role of the funding source

The funding sources had no involvement in the conduct of the research and preparation of the article, in study design, in the collection, analysis and interpretation of data; in the writing of the report; and in the decision to submit the article for publication.

## Declaration of competing interest

The authors have no conflicts of interest to declare in relation to the work presented in this paper.

## Appendix A Supplementary data

Supplementary material Image 1

## References

[bb0005] Allardyce J., McCreadie R., Morrison G., van Os J. (2007). Do symptom dimensions or categorical diagnoses best discriminate between known risk factors for psychosis?. Soc. Psychiatry Psychiatr. Epidemiol..

[bb0010] Allen D.N., Strauss G.P., Barchard K.A., Vertinski M., Carpenter W.T., Buchanan R.W. (2013). Differences in developmental changes in academic and social premorbid adjustment between males and females with schizophrenia. Schizophr. Res..

[bb0015] Arango C., Fraguas D., Parellada M. (2014). Differential neurodevelopmental trajectories in patients with early-onset bipolar and schizophrenia disorders. Schizophr. Bull..

[bb0020] Ayesa-Arriola R., Setién-Suero E., Neergaard K.D., Ferro A., Fatjó-Vilas M., Ríos-Lago M., Otero S., Rodríguez-Sánchez J.M., Crespo-Facorro B. (2016). Evidence for trait related theory of mind impairment in first episode psychosis patients and its relationship with processing speed: a 3 year follow-up study. Front. Psychol..

[bb0025] Brill N., Reichenberg A., Weiser M., Rabinowitz J. (2008). Validity of the premorbid adjustment scale. Schizophr. Bull..

[bb0030] Brill N., Levine S.Z., Reichenberg A., Lubin G., Weiser M., Rabinowitz J. (2009). Pathways to functional outcomes in schizophrenia: the role of premorbid functioning, negative symptoms and intelligence. Schizophr. Res..

[bb0035] Bucci P., Galderisi S., Mucci A., Rossi A., Rocca P., Bertolino A., Aguglia E., Amore M., Andriola I., Bellomo A., Biondi M., Cuomo A., Dell’Osso L., Favaro A., Gambi F., Giordano G., Girardi P., Marchesi C., Monteleone P., Montemagni C., Niolu C., Oldani L., Pacitti F., Pinna F., Roncone R., Vita A., Zeppegno P., Maj M. (2018). Italian network for research on psychoses. Premorbid academic and social functioning in patients with schizophrenia and its associations with negative symptoms and cognition. Acta Psychiatr. Scand..

[bb0040] Bucci P., Galderisi S., Mucci A., Rossi A., Rocca P., Bertolino A., Aguglia E., Amore M., Andriola I., Bellomo A., Biondi M., Cuomo A., Dell’osso L., Favaro A., Gambi F., Giordano G.M., Girardi P., Marchesi C., Monteleone P., Montemagni C., Niolu C., Oldani L., Pacitti F., Pinna F., Roncone R., Vita A., Zeppegno P., Maj M., Patriarca S., Pietrafesa D., Aiello C., Longo L., Barone M., Romano R., Atti A.R., Barlati S., Deste G., Valsecchi P., Carpiniello B., Tusconi M., Puddu L., Signorelli M.S., Cannavò D., Minutolo G., Corbo M., Montemitro C., Baroni G., Altamura M., La Montagna M., Carnevale R., Murri M.B., Calcagno P., Bugliani M., Pizziconi G., Logozzo F., Rossi R., Giusti L., Salza A., Malavolta M., Orsenigo G., Grassi S., De Bartolomeis A., Gramaglia C., Gattoni E., Gambaro E., Tenconi E., Ferronato L., Collantoni E., Tonna M., Ossola P., Gerra M.L., Carmassi C., Cremone I.M., Carpita B., Buzzanca A., Girardi N., Frascarelli M., Del Casale A., Comparelli A., Corigliano V., Siracusano A., Di Lorenzo G., Ribolsi M., Corrivetti G., Bartoli L., Del Buono G., Fagiolini A., Bolognesi S., Goracci A., Mancini I., Bava I., Cardillo S. (2018). Premorbid academic and social functioning in patients with schizophrenia and its associations with negative symptoms and cognition. Acta Psychiatr. Scand..

[bb0045] Cannon-Spoor H.E., Potkin S.G., Wyatt R.J. (1982). Measurement of premorbid adjustment in chronic schizophrenia. Schizophr. Bull..

[bb0050] Caspi A., Houts R.M., Ambler A., Danese A., Elliott M.L., Hariri A., Harrington H.L., Hogan S., Poulton R., Ramrakha S., Rasmussen L.J.H., Reuben A., Richmond-Rakerd L., Sugden K., Wertz J., Williams B.S., Moffitt T.E. (2020). Longitudinal assessment of mental health disorders and comorbidities across 4 decades among participants in the Dunedin birth cohort study. JAMA Netw. Open.

[bb0055] Chang W.C., Tang J.Y.M., Hui C.L.M., Wong G.H.Y., Chan S.K.W., Lee E.H.M., Chen E.Y.H. (2013). The relationship of early premorbid adjustment with negative symptoms and cognitive functions in first-episode schizophrenia: a prospective three-year follow-up study. Psychiatry Res..

[bb0060] Chang W.C., Lau C.F.C., Chan S.S.I., Hui C.L.M., Chan S.K.W., Lee E.H.M., Lin J., Chen E.Y.H. (2016). Premorbid, clinical and cognitive correlates of primary negative symptoms in first-episode psychosis. Psychiatry Res..

[bb0065] Cohen A.S., Brown L.A., Minor K.S. (2010). The psychiatric symptomatology of deficit schizophrenia: a meta-analysis. Schizophr. Res..

[bb0070] Cole V.T., Apud J.A., Weinberger D.R., Dickinson D. (2012). Using latent class growth analysis to form trajectories of premorbid adjustment in schizophrenia. J. Abnorm. Psychol..

[bb0075] CORDIS (2019).

[bb0080] Culbreth A.J., Moran E.K., Barch D.M. (2018). Effort-cost decision-making in psychosis and depression: could a similar behavioral deficit arise from disparate psychological and neural mechanisms?. Psychol. Med..

[bb0085] Demjaha A., MacCabe J.H., Murray R.M. (2012). How genes and environmental factors determine the different neurodevelopmental trajectories of schizophrenia and bipolar disorder. Schizophr. Bull..

[bb0090] Demjaha A., Valmaggia L., Stahl D., Byrne M., McGuire P. (2012). Disorganization/cognitive and negative symptom dimensions in the at-risk mental state predict subsequent transition to psychosis. Schizophr. Bull..

[bb0095] Di Forti M., Quattrone D., Freeman T.P., Tripoli G., Gayer-Anderson C., Quigley H., Rodriguez V., Jongsma H.E., Ferraro L., La Cascia C., La Barbera D., Tarricone I., Berardi D., Szöke A., Arango C., Tortelli A., Velthorst E., Bernardo Miguel, Del-Ben C.M., Menezes P.R., Selten J.P., Jones P.B., Kirkbride J.B., Rutten B.P., de Haan L., Sham P.C., van Os J., Lewis C.M., Lynskey M., Morgan C., Murray R.M., Amoretti S., Arrojo M., Baudin G., Beards S., Bernardo Miquel, Bobes J., Bonetto C., Cabrera B., Carracedo A., Charpeaud T., Costas J., Cristofalo D., Cuadrado P., Díaz-Caneja C.M., Ferchiou A., Franke N., Frijda F., García Bernardo E., Garcia-Portilla P., González E., Hubbard K., Jamain S., Jiménez-López E., Leboyer M., López Montoya G., Lorente-Rovira E., Marcelino Loureiro C., Marrazzo G., Martínez C., Matteis M., Messchaart E., Moltó M.D., Nacher J., Olmeda M.S., Parellada M., González Peñas J., Pignon B., Rapado M., Richard J.R., Rodríguez Solano J.J., Roldán Díaz L., Ruggeri M., Sáiz P.A., Sánchez E., Sanjuán J., Sartorio C., Schürhoff F., Seminerio F., Shuhama R., Sideli L., Stilo S.A., Termorshuizen F., Tosato S., Tronche A.M., van Dam D., van der Ven E. (2019). The contribution of cannabis use to variation in the incidence of psychotic disorder across Europe (EU-GEI): a multicentre case-control study. Lancet Psychiatry.

[bb0100] Díaz-Caneja C.M., Pina-Camacho L., Rodríguez-Quiroga A., Fraguas D., Parellada M., Arango C. (2015). Predictors of outcome in early-onset psychosis: a systematic review. NPJ Schizophr..

[bb0105] Dickinson D., Ramsey M.E., Gold J.M. (2007). Overlooking the obvious: a meta-analytic comparison of digit symbol coding tasks and other cognitive measures in schizophrenia. Arch. Gen. Psychiatry.

[bb0110] Drake R.J., Addington J., Viswanathan A.C., Lewis S.W., Cotter J., Yung A.R., Abel K.M. (2016). How age and gender predict illness course in a first-episode nonaffective psychosis cohort. J. Clin. Psychiatry.

[bb0115] van Os J., Rutten B.P., Myin-Germeys I., Delespaul P., Viechtbauer W., van Zelst C., Bruggeman R., Reininghaus U., Morgan C., Murray R.M., Di Forti M., McGuire P., Valmaggia L.R., Kempton M.J., Gayer-Anderson C., Hubbard K., Beards S., Stilo S.A., Onyejiaka A., Bourque F., Modinos G., Tognin S., Calem M., O’Donovan M.C., Owen M.J., Holmans P., Williams N., Craddock N., Richards A., Humphreys I., Meyer-Lindenberg A., Leweke F.M., Tost H., Akdeniz C., Rohleder C., Bumb J.M., Schwarz E., Alptekin K., Üçok A., Saka M.C., Atbasoglu E.C., Gülöksüz S., Gumus-Akay G., Cihan B., Karadag H., Soygür H., Cankurtaran E.S., Ulusoy S., Akdede B., Binbay T., Ayer A., Noyan H., Karadayi G., Akturan E., Ulas H., Arango C., Parellada M., Bernardo M., Sanjuán J., Bobes J., Arrojo M., Santos J.L., Cuadrado P., Rodríguez Solano J.J., Carracedo A., García Bernardo E., Roldán L., López G., Cabrera B., Cruz S., Díaz Mesa E.M., Pouso M., Jiménez E., Sánchez T., Rapado M., González E., Martínez C., Sánchez E., Olmeda M.S., de Haan L., Velthorst E., van der Gaag M., Selten J.-P., van Dam D., van der Ven E., van der Meer F., Messchaert E., Kraan T., Burger N., Leboyer M., Szoke A., Schürhoff F., Llorca P.-M., Jamain S., Tortelli A., Frijda F., Vilain J., Galliot A.-M., Baudin G., Ferchiou A., Richard J.-R., Bulzacka E., Charpeaud T., Tronche A.-M., De Hert M., van Winkel R., Decoster J., Derom C., Thiery E., Stefanis N.C., Sachs G., Aschauer H., Lasser I., Winklbaur B., Schlögelhofer M., Riecher-Rössler A., Borgwardt S., Walter A., Harrisberger F., Smieskova R., Rapp C., Ittig S., Soguel-dit-Piquard F., Studerus E., Klosterkötter J., Ruhrmann S., Paruch J., Julkowski D., Hilboll D., Sham P.C., Cherny S.S., Chen E.Y.H., Campbell D.D., Li M., Romeo-Casabona C.M., Emaldi Cirión A., Urruela Mora A., Jones P., Kirkbride J., Cannon M., Rujescu D., Tarricone I., Berardi D., Bonora E., Seri M., Marcacci T., Chiri L., Chierzi F., Storbini V., Braca M., Minenna M.G., Donegani I., Fioritti A., La Barbera D., La Cascia C.E., Mulè A., Sideli L., Sartorio R., Ferraro L., Tripoli G., Seminerio F., Marinaro A.M., McGorry P., Nelson B., Amminger G.P., Pantelis C., Menezes P.R., Del-Ben C.M., Gallo Tenan S.H., Shuhama R., Ruggeri M., Tosato S., Lasalvia A., Bonetto C., Ira E., Nordentoft M., Krebs M.-O., Barrantes-Vidal N., Cristóbal P., Kwapil T.R., Brietzke E., Bressan R.A., Gadelha A., Maric N.P., Andric S., Mihaljevic M., Mirjanic T., European Network of National Networks studying Gene-Environment Interactions in Schizophrenia (EU-GEI) (2014). Identifying gene-environment interactions in schizophrenia: contemporary challenges for integrated, large-scale investigations. Schizophr. Bull..

[bb0120] Ferraro L., Russo M., O’Connor J., Wiffen B.D.R., Falcone M.A., Sideli L., Gardner-Sood P., Stilo S., Trotta A., Dazzan P., Mondelli V., Taylor H., Friedman B., Sallis H., La Cascia C., La Barbera D., David A.S., Reichenberg A., Murray R.M., Di Forti M. (2013). Cannabis users have higher premorbid IQ than other patients with first onset psychosis. Schizophr. Res..

[bb0125] Ferraro L., Cascia C.La, Quattrone D., Sideli L., Matranga D., Capuccio V., Tripoli G., Gayer-Anderson C., Morgan C., Sami M.B., Sham P., de Haan L., Velthorst E., Jongsma H.E., Kirkbride J.B., Rutten B.P.F., Richards A.L., Roldan L., Arango C., Bernardo M., Bobes J., Sanjuan J., Santos J.L., Arrojo M., Tarricone I., Tortelli A., SzÃ¶ke A., Del-Ben C.M., Selten J.-P., Lynskey M., Jones P.B., Os J.Van, Barbera D.La, Murray R.M., Forti M.Di (2019). Premorbid adjustment and {IQ} in patients with first-episode psychosis: a multisite case-control study of their relationship with cannabis use. Schizophr. Bull..

[bb0130] Forbes N.F., Carrick L.A., McIntosh A.M., Lawrie S.M. (2009). Working memory in schizophrenia: a meta-analysis. Psychol. Med..

[bb0135] Freeman D., Startup H., Dunn G., Cernis E., Wingham G., Pugh K., Cordwell J., Mander H., Kingdon D. (2014). Understanding jumping to conclusions in patients with persecutory delusions: working memory and intolerance of uncertainty. Psychol. Med..

[bb0140] Fry A.F., Hale S. (2000). Relationships among processing speed, working memory, and fluid intelligence in children. Biol. Psychol..

[bb0145] Galderisi S., Bucci P., Üçok A., Peuskens J. (2012). No gender differences in social outcome in patients suffering from schizophrenia. Eur. Psychiatry.

[bb0150] Gayer-Anderson C., Jongsma H.E., Di Forti M., Quattrone D., Velthorst E., de Haan L., Selten J.P., Szöke A., Llorca P.M., Tortelli A., Arango C., Bobes J., Bernardo M., Sanjuán J., Santos J.L., Arrojo M., Parellada M., Tarricone I., Berardi D., Ruggeri M., Lasalvia A., Ferraro L., La Cascia C., La Barbera D., Menezes P.R., Del-Ben C.M., Hubbard K., Beards S., Reininghaus U., Tripoli G., Stilo S.A., Roldán L., López G., Matteis M., Rapado M., González E., Martínez C., Cuadrado P., Solano J.J.R., Carracedo A., Costas J., Bernardo E.G., Sánchez E., Olmeda M.S., Cabrera B., Lorente-Rovira E., Garcia-Portilla P., Jiménez-López E., Franke N., van Dam D., Termorshuizen F., van der Ven E., Messchaart E., Leboyer M., Schürhoff F., Baudin G., Ferchiou A., Pignon B., Jamain S., Richard J.R., Charpeaud T., Tronche A.M., Frijda F., Sideli L., Seminerio F., Sartorio C., Marrazzo G., Loureiro C.M., Shuhama R., Tosato S., Bonetto C., Cristofalo D., Rutten B.P., van Os J., Jones P.B., Murray R.M., Kirkbride J.B., Morgan C. (2020). The EUropean network of National Schizophrenia Networks Studying Gene-Environment Interactions (EU-GEI): incidence and first-episode case-control programme. Soc. Psychiatry Psychiatr. Epidemiol..

[bb0155] Gisselgård J., Anda L.G., Brønnick K., Langeveld J., Ten Velden Hegelstad W., Joa I., Johannessen J.O., Larsen T.K. (2014). Verbal working memory deficits predict levels of auditory hallucination in first-episode psychosis. Schizophr. Res..

[bb0160] de Gracia Dominguez M., Viechtbauer W., Simons C.J.P., van Os J., Krabbendam L. (2009). Are psychotic psychopathology and neurocognition orthogonal? A systematic review of their associations. Psychol. Bull..

[bb0165] Grau N., Rubio-Abadal Elena, Usall Judith, Barajas Ana, Butjosa A., Dolz Montserrat, Baños Iris, Sánchez Bernardo, Rodríguez M.J., Peláez T., Sammut Stephanie, Carlson Janina, Huerta-Ramos E., Ochoa Susana, Araya S., Arranz B., Arteaga M., Asensio R., Autonell J., Baños I., Bañuelos M., Barajas A., Barceló M., Blanc M., Borrás M., Busquets E., Carlson J., Carral V., Castro M., Corbacho C., Cor-omina M., Dachs I., DeMiquel L., Dolz M., Domenech M.D., Elias M., Espezel I., Falo E., Fargas A., Foix A., Fusté M., Godrid M., Gómez D., González O., Granell L., Gumà L., Haro J.M., Herrera S., Huerta E., Lacasa F., Mas N., Martí L., Martínez R., Matalí J., Miñambres A., Muñoz D., Muñoz V., Nogueroles R., Núñez M., Ochoa S., Ortiz J., Pardo M., Planella M., Pelaez T., Peruzzi S., Portos J., Rivero S., Rodriguez M.J., Rubio-Abadal E., Sammut S., Sánchez M., Sánchez B., Serrano E., Solís C., Stephan-Otto C., Tabuenca P., Teba S., Torres A., Urbano D., Usall J., Vilaplana M., Villalta V. (2016). Influence of cognition, premorbid adjustment and psychotic symptoms on psycho-social functioning in first-episode psychosis. Psychiatry Res..

[bb0170] Green R.E.A., Melo B., Christensen B., Ngo L.-A., Monette G., Bradbury C. (2008). Measuring premorbid IQ in traumatic brain injury: an examination of the validity of the wechsler test of adult Reading (WTAR). J. Clin. Exp. Neuropsychol..

[bb0175] Guerra A., Fearon P., Sham P., Jones P., Lewis S., Mata I., Murray R. (2002). The relationship between predisposing factors, premorbid function and symptom dimensions in psychosis: an integrated approach. Eur. Psychiatry.

[bb0180] Horton L.E., Tarbox S.I., Olino T.M., Haas G.L. (2015). Trajectories of premorbid childhood and adolescent functioning in schizophrenia-spectrum psychoses: a first-episode study. Psychiatry Res..

[bb0185] IBM Corporation (2017).

[bb0190] Jenkins L.M., Bodapati A.S., Sharma R.P., Rosen C. (2018). Working memory predicts presence of auditory verbal hallucinations in schizophrenia and bipolar disorder with psychosis. J. Clin. Exp. Neuropsychol..

[bb0195] Jongsma H.E., Gayer-Anderson C., Lasalvia A., Quattrone D., Mulè A., Szöke A., Selten J.P., Turner C., Arango C., Tarricone I., Berardi D., Tortelli A., Llorca P.M., De Haan L., Bobes J., Bernardo M., Sanjuán J., Santos J.L., Arrojo M., Del-Ben C.M., Menezes P.R., Murray R.M., Rutten B.P., Jones P.B., Van Os J., Morgan C., Kirkbride J.B., Reininghaus U., Di Forti M., Hubbard K., Beards S., Stilo S.A., Tripoli G., Parellada M., Cuadrado P., Solano J.J.R., Carracedo A., Bernardo E.G., Roldán L., López G., Cabrera B., Lorente-Rovira E., Garcia-Portilla P., Costas J., Jiménez-López E., Matteis M., Rapado M., González E., Martínez C., Sánchez E., Olmeda M.S., Franke N., Velthorst E., Termorshuizen F., Van Dam D., Van Der Ven E., Messchaart E., Leboyer M., Schürhoff F., Jamain S., Frijda F., Baudin G., Ferchiou A., Pignon B., Richard J.R., Charpeaud T., Tronche A.M., La Barbera D., La Cascia C., Marrazzo G., Sideli L., Sartorio C., Ferraro L., Seminerio F., Loureiro C.M., Shuhama R., Ruggeri M., Tosato S., Bonetto C., Cristofalo D. (2018). Treated incidence of psychotic disorders in the multinational EU-GEI study. JAMA Psychiatry.

[bb0200] Koenen K.C., Moffitt T.E., Roberts A.L., Martin L.T., Kubzansky L., Harrington H., Poulton R., Caspi A. (2009). Childhood IQ and adult mental disorders: a test of the cognitive reserve hypothesis. Am. J. Psychiatry.

[bb0205] Kotov R., Jonas K.G., Carpenter W.T., Dretsch M.N., Eaton N.R., Forbes M.K., Forbush K.T., Hobbs K., Reininghaus U., Slade T., South S.C., Sunderland M., Waszczuk M.A., Widiger T.A., Wright A., Zald D.H., Krueger R.F., Watson D., Kotov R., Jonas K.G., Carpenter W.T., Dretsch M.N., Eaton N.R., Forbes M.K., Forbush K.T., Hobbs K., Reininghaus U., Slade T., South S.C., Sunderland M., Waszczuk M.A., Widiger T.A., Wright A., Zald D.H., Krueger R.F., Watson D. (2020). Validity and utility of Hierarchical Taxonomy of Psychopathology (HiTOP): I. Psychosis superspectrum. World Psychiatry.

[bb0210] Kravariti E., Russo M., Vassos E., Morgan K., Fearon P., Zanelli J.W., Demjaha A., Lappin J.M., Tsakanikos E., Dazzan P., Morgan C., Doody G.A., Harrison G., Jones P.B., Murray R.M., Reichenberg A. (2012). Linear and non-linear associations of symptom dimensions and cognitive function in first-onset psychosis. Schizophr. Res..

[bb0215] Kravariti E., Demjaha A., Zanelli J., Ibrahim F., Wise C., MacCabe J.H., Reichenberg A., Pilecka I., Morgan K., Fearon P., Morgan C., Doody G.A., Donoghue K., Jones P.B., Kaçar A.S., Dazzan P., Lappin J., Murray R.M. (2019). Neuropsychological function at first episode in treatment-resistant psychosis: findings from the ÆsOP-10 study. Psychol. Med..

[bb0220] Laloyaux J., Della Libera C., Larøi F. (2018). Source flexibility in schizophrenia: specificity and role in auditory hallucinations. Cogn. Neuropsychiatry.

[bb0225] Larsen T.K., Friis S., Haahr U., Johannessen J.O., Melle I., Opjordsmoen S., Rund B.R., Simonsen E., Vaglum P., McGlashan T.H. (2004). Premorbid adjustment in first-episode non-affective psychosis: distinct patterns of pre-onset course. Br. J. Psychiatry.

[bb0230] Laursen T.M., Agerbo E., Pedersen C.B. (2009). Bipolar disorder, schizoaffective disorder, and schizophrenia overlap: a new comorbidity index. J. Clin. Psychiatry.

[bb0235] Lee J., Park S. (2005). Working memory impairments in schizophrenia: a meta-analysis. J. Abnorm. Psychol..

[bb0240] Linscott R.J., van Os J. (2010). Systematic reviews of categorical versus continuum models in psychosis: evidence for discontinuous subpopulations underlying a psychometric continuum. implications for DSM-V, DSM-VI, and DSM-VII. Annu. Rev. Clin. Psychol..

[bb0245] MacBeth A., Gumley A. (2008). Premorbid adjustment, symptom development and quality of life in first episode psychosis: a systematic review and critical reappraisal. Acta Psychiatr. Scand..

[bb0250] Mallett R., Leff J., Bhugra D., Pang D., Zhao J.H. (2002). Social environment, ethnicity and schizophrenia. a case-control study. Soc. Psychiatry Psychiatr. Epidemiol..

[bb0255] van Mastrigt S., Addington J. (2002). Assessment of premorbid function in first-episode schizophrenia: modifications to the premorbid adjustment scale. J. Psychiatry Neurosci..

[bb0260] Mathias S.R., Knowles E.E.M., Barrett J., Leach O., Buccheri S., Beetham T., Blangero J., Poldrack R.A., Glahn D.C. (2017). The processing-speed impairment in psychosis is more than just accelerated aging. Schizophr. Bull..

[bb0265] McGuffin P., Farmer A., Harvey I. (1991). A polydiagnostic application of operational criteria in studies of psychotic illness: development and reliability of the OPCRIT system. Arch. Gen. Psychiatry.

[bb0270] Minor K.S., Lysaker P.H. (2014). Necessary, but not sufficient: links between neurocognition, social cognition, and metacognition in schizophrenia are moderated by disorganized symptoms. Schizophr. Res..

[bb0275] Minor K.S., Marggraf M.P., Davis B.J., Luther L., Vohs J.L., Buck K.D., Lysaker P.H. (2015). Conceptual disorganization weakens links in cognitive pathways: disentangling neurocognition, social cognition, and metacognition in schizophrenia. Schizophr. Res..

[bb0280] Mollon J., David A.S., Zammit S., Lewis G., Reichenberg A. (2018). Course of cognitive development from infancy to early adulthood in the psychosis spectrum. JAMA Psychiatry.

[bb0285] Monte R.C., Goulding S.M., Compton M.T. (2008). Premorbid functioning of patients with first-episode nonaffective psychosis: a comparison of deterioration in academic and social performance, and clinical correlates of premorbid adjustment scale scores. Schizophr. Res..

[bb0290] Morgan V.A., Castle D.J., Jablensky A.V. (2008). Do women express and experience psychosis differently from men? Epidemiological evidence from the australian National Study of low prevalence (Psychotic) disorders. Aust. N. Z. J. Psychiatry.

[bb0295] Morita K., Miura K., Fujimoto M., Yamamori H., Yasuda Y., Kudo N., Azechi H., Okada N., Koshiyama D., Ikeda M., Kasai K., Hashimoto R. (2019). Eye movement abnormalities and their association with cognitive impairments in schizophrenia. Schizophr. Res..

[bb0300] Murphy D., Vallières F., Murphy J., McElroy E., Hyland P. (2020). Risk factors associated with general and specific dimensions of psychosis in a nationally representative sample of adults from the United States. Psychosis.

[bb0305] Murray R.M., Castle D.J. (1991). The neurodevelopmental basis of sex differences in schizophrenia. Psychol. Med..

[bb0310] Murray R.M., Sham P., Van Os J., Zanelli J., Cannon M., McDonald C. (2004). A developmental model for similarities and dissimilarities between schizophrenia and bipolar disorder. Schizophr. Res..

[bb0315] Murray R.M., Bhavsar V., Tripoli G., Howes O. (2017). 30 years on: how the neurodevelopmental hypothesis of schizophrenia morphed into the developmental risk factor model of psychosis. Schizophr. Bull..

[bb0320] Neisser U., Boodoo G., Bouchard T.J., Boykin A.W., Brody N., Ceci S.J., Halpern D.F., Loehlin J.C., Perloff R., Sternberg R.J., Urbina S. (1996). Intelligence: knowns and unknowns. Am. Psychol..

[bb0325] van Os J., Rutten B.P., Poulton R. (2008). Gene-environment interactions in schizophrenia: review of epidemiological findings and future directions. Schizophr. Bull..

[bb0330] Owen M.J., O’Donovan M.C. (2017). Schizophrenia and the neurodevelopmental continuum:evidence from genomics. World Psychiatry.

[bb0335] Parellada M., Gomez-Vallejo S., Burdeus M., Arango C. (2017). Developmental differences between schizophrenia and bipolar disorder. Schizophr. Bull..

[bb9000] Payá B., Rodríguez-Sánchez J.M., Otero S., Muñoz P., Castro-Fornieles J., Parellada M., Gonzalez-Pinto A., Soutullo C., Baeza I., Rapado-Castro M., Sáenz-Herrero M., Moreno D., Arango C. (2013 May). Premorbid impairments in early-onset psychosis: differences between patients with schizophrenia and bipolar disorder. Schizophr Res..

[bb0340] Petruzzelli M.G., Margari L., Bosco A., Craig F., Palumbi R., Margari F. (2018). Early onset first episode psychosis: dimensional structure of symptoms, clinical subtypes and related neurodevelopmental markers. Eur. Child Adolesc. Psychiatry.

[bb0345] Pope L.G., Manseau M.W., Kelley M.E., Compton M.T. (2021). Symptomatology and neurocognition among first-episode psychosis patients with and without cannabis use in the three months prior to first hospitalization. Schizophr. Res..

[bb0350] Quattrone D., Di Forti M., Gayer-Anderson C., Ferraro L., Jongsma H.E., Tripoli G., La Cascia C., La Barbera D., Tarricone I., Berardi D., Szöke A., Arango C., Lasalvia A., Tortelli A., Llorca P.M., De Haan L., Velthorst E., Bobes J., Bernardo M., Sanjuán J., Santos J.L., Arrojo M., Del-Ben C.M., Menezes P.R., Selten J.P., Jones P.B., Kirkbride J.B., Richards A.L., O’Donovan M.C., Sham P.C., Vassos E., Rutten B.P., Van Os J., Morgan C., Lewis C.M., Murray R.M., Reininghaus U., Hubbard K., Beards S., Stilo S.A., Parellada M., Cuadrado P., Solano J.J.R., Carracedo A., Bernardo E.G., Roldan L., Lopez G., Cabrera B., Lorente-Rovira E., Garcia-Portilla P., Costas J., Jimenez-Lopez E., Matteis M., Rapado M., Gonzalez E., Martinez C., Sanchez E., Olmeda M.S., Franke N., Termorshuizen F., Van Dam D., Van Der Ven E., Messchaart E., Leboyer M., Schürhoff F., Jamain S., Baudin G., Ferchiou A., Pignon B., Richard J.R., Charpeaud T., Tronche A.M., Frijda F., Marrazzo G., Sideli L., Sartorio C., Seminerio F., Loureiro C.M., Shuhama R., Ruggeri M., Tosato S., Bonetto C., Cristofalo D. (2019). Transdiagnostic dimensions of psychopathology at first episode psychosis: findings from the multinational EU-GEI study. Psychol. Med..

[bb0355] Quattrone D., Ferraro L., Tripoli G., La Cascia C., Quigley H., Quattrone A., Jongsma H.E., Del Peschio S., Gatto G., Gayer-Anderson C., Jones P.B., Kirkbride J.B., La Barbera D., Tarricone I., Berardi D., Tosato S., Lasalvia A., Szöke A., Arango C., Di Forti M. (2020). Daily use of high-potency cannabis is associated with more positive symptoms in first-episode psychosis patients: the EU-GEI case-control study. Psychol. Med..

[bb0360] Rabinowitz J., De Smedt G., Harvey P.D., Davidson M. (2002). Relationship between premorbid functioning and symptom severity as assessed at first episode of psychosis. Am. J. Psychiatry.

[bb0365] Rabinowitz J., Haim R., Reichenberg A., Weiser M., Kaplan Z., Davidson M., Häfner H. (2005). Association between functioning in adolescence prior to first admission for schizophrenia and affective disorders and patterns of hospitalizations thereafter. Schizophr. Res..

[bb0370] Rabinowitz J., Harvey P.D., Eerdekens M., Davidson M. (2006). Premorbid functioning and treatment response in recent-onset schizophrenia. Br. J. Psychiatry.

[bb0375] Rabinowitz J., Levine S.Z., Brill N., Bromet E.J. (2007). The premorbid adjustment scale structured interview (PAS-SI): preliminary findings. Schizophr. Res..

[bb0380] Reininghaus U., Böhnke J.R., Hosang G., Farmer A., Burns T., McGuffin P., Bentall R.P. (2016). Evaluation of the validity and utility of a transdiagnostic psychosis dimension encompassing schizophrenia and bipolar disorder. Br. J. Psychiatry.

[bb0385] Reise S.P., Morizot J., Hays R.D. (2007). The role of the bifactor model in resolving dimensionality issues in health outcomes measures. Qual. Life Res..

[bb0390] Riecher-Rössler A., Häfner H. (2000). Gender aspects in schizophrenia: bridging the border between social and biological psychiatry. Acta Psychiatr. Scand. Suppl..

[bb0395] Ringen P.A., Nesvåg R., Helle S., Lagerberg T.V., Lange E.H., Loberg E.M., Agartz I., Andreassen O.A., Melle I. (2016). Premorbid cannabis use is associated with more symptoms and poorer functioning in schizophrenia spectrum disorder. Psychol. Med..

[bb0400] Rodríguez-Sánchez J.M., Crespo-Facorro B., González-Blanch C., Pérez-Iglesias R., Vázquez-Barquero J.L. (2007). Cognitive dysfunction in first-episode psychosis: the processing speed hypothesis. Br. J. Psychiatry.

[bb0405] Scheiber C., Chen H., Kaufman A.S., Weiss L.G. (2017). How much does WAIS-IV perceptual reasoning decline across the 20 to 90-year lifespan when processing speed is Controlled?. Appl. Neuropsychol. Adult.

[bb0410] Seddon J.L., Birchwood M., Copello A., Everard L., Jones P.B., Fowler D., Amos T., Freemantle N., Sharma V., Marshall M., Singh S.P. (2016). Cannabis use is associated with increased psychotic symptoms and poorer psychosocial functioning in first-episode psychosis: a report from the UK national EDEN study. Schizophr. Bull..

[bb0415] Seeman M.V. (1997). Psychopathology in women and men: focus on female hormones. Am. J. Psychiatry.

[bb0420] Sheffield J.M., Karcher N.R., Barch D.M. (2018). Cognitive deficits in psychotic disorders: a lifespan perspective. Neuropsychol. Rev..

[bb0425] Silver H., Feldman P., Bilker W., Gur R.C. (2003). Working memory deficit as a core neuropsychological dysfunction in schizophrenia. Am. J. Psychiatry.

[bb0430] Silverstein S.M., Keane B.P. (2011). Perceptual organization impairment in schizophrenia and associated brain mechanisms: review of research from 2005 to 2010. Schizophr. Bull..

[bb0435] Singh S.P., Cooper J.E., Fisher H.L., Tarrant C.J., Lloyd T., Banjo J., Corfe S., Jones P. (2005). Determining the chronology and components of psychosis onset: the Nottingham onset schedule (NOS). Schizophr. Res..

[bb0440] Stouten L.H., Veling W., Laan W., van der Helm M., van der Gaag M. (2017). Psychosocial functioning in first-episode psychosis and associations with neurocognition, social cognition, psychotic and affective symptoms. Early Interv. Psychiatry.

[bb0445] Strauss G.P., Allen D.N., Miski P., Buchanan R.W., Kirkpatrick B., Carpenter W.T. (2012). Differential patterns of premorbid social and academic deterioration in deficit and nondeficit schizophrenia. Schizophr. Res..

[bb0450] Tarbox S.I., Brown L.H., Haas G.L. (2012). Diagnostic specificity of poor premorbid adjustment: comparison of schizophrenia, schizoaffective disorder, and mood disorder with psychotic features. Schizophr. Res..

[bb0455] Tarbox S.I., Addington J., Cadenhead K.S., Cannon T.D., Cornblatt B.A., Perkins D.O., Seidman L.J., Tsuang M.T., Walker E.F., Heinssen R., McGlashan T.H., Woods S.W. (2013). Premorbid functional development and conversion to psychosis in clinical high-risk youths. Dev. Psychopathol..

[bb0460] Torrent C., Reinares M., Martinez-Arán A. (2018). Affective versus non-affective first episode psychoses: a longitudinal study. J. Affect. Disord..

[bb0465] Trotta A., Murray R.M., MacCabe J.H. (2015). Do premorbid and post-onset cognitive functioning differ between schizophrenia and bipolar disorder? A systematic review and meta-analysis. Psychol. Med..

[bb0470] Upthegrove R., Marwaha S., Birchwood M. (2017). Depression and schizophrenia: cause, consequence, or trans-diagnostic issue?. Schizophr. Bull..

[bb0475] Van Os J., Gilvarry C., Bale R., Van Horn E., Tattan T., White I., Murray R. (1999). A comparison of the utility of dimensional and categorical representations of psychosis. Psychol. Med..

[bb0480] Velthorst E., Levine S.Z., Henquet C., De Haan L., Van Os J., Myin-Germeys I., Reichenberg A. (2013). To cut a short test even shorter: reliability and validity of a brief assessment of intellectual ability in schizophrenia - a control-case family study. Cogn. Neuropsychiatry.

[bb0485] Ventura J., Thames A.D., Wood R.C., Guzik L.H., Hellemann G.S. (2010). Disorganization and reality distortion in schizophrenia: a meta-analysis of the relationship between positive symptoms and neurocognitive deficits. Schizophr. Res..

[bb0490] Williams J., Farmer A.E., Ackenheil M., Kaufmann C.A., McGuffin P., Group, T.O.R.R (1996). A multicentre inter-rater reliability study using the OPCRIT computerized diagnostic system. Psychol. Med..

[bb0495] World Health Organization (1992). The ICD-10 classification of mental and behavioural disorders: clinical descriptions and diagnostic guidelines [WWW document]. https://www.who.int/classifications/icd/en/bluebook.pdf.

[bb0500] Yuan K., Steedle J., Shavelson R., Alonzo A., Oppezzo M. (2006). Working memory, fluid intelligence, and science learning. Educ. Res. Rev..

[bb0505] Zanelli J., Reichenberg A., Morgan K., Fearon P., Kravariti E., Dazzan P., Morgan C., Zanelli C., Demjaha A., Jones P.B., Doody G.A., Kapur S., Murray R.M. (2010). Specific and generalized neuropsychological deficits: a comparison of patients with various first-episode psychosis presentations. Am. J. Psychiatry.

[bb0510] Zhu C., Sun X., So S.H.Wai (2018). Associations between belief inflexibility and dimensions of delusions: a meta-analytic review of two approaches to assessing belief flexibility. Br. J. Clin. Psychol..

