# Supplementary material for: The relationship of symptom dimensions with premorbid adjustment and cognitive characteristics at first episode psychosis: Findings from the EU-GEI study
Source: Schizophr Res. 2021 Oct;236:69–79. doi: 10.1016/j.schres.2021.08.008 (PMC8473991; doi:10.1016/j.schres.2021.08.008)
Supplement: Supplementary file 1 — Supplementary material [file mmc1.docx]

SUPPLEMENTARY MATERIAL

[*Summary of statistical analysis from our previous studies* 2](#_Toc79487682)

[**Supplementary Table 1. ICD-10 Diagnoses** 2](#_Toc79487683)

[*Pearson Correlations Between Symptom Dimensions, IQ, and Premorbid Characteristics* 3](#_Toc79487684)

[**Supplementary Table 2. Pearson Correlations Between Symptom Dimensions. IQ. and Premorbid Characteristics** 3](#_Toc79487685)

[*The role of cannabis use* 4](#_Toc79487686)

[**Supplementary-Figure 1. Grouped Scatter Mean of Predicted Value for Positive Symptoms by Processing Speed by Frequency of cannabis Use** 5](#_Toc79487687)

[*The effect of the other predictors* 6](#_Toc79487688)

[**Supplementary Table 3. Parameter Estimates in the Predictive Model for Symptom Dimensions by PSF, PAF and IQ** 7](#_Toc79487689)

[*DUP and AP treatment influence on the relationship between symptom dimensions and cognitive and premorbid adjustment* 10](#_Toc79487690)

[References 11](#_Toc79487691)

*Summary of statistical analysis from our previous studies*

Multidimensional item response modelling was used in Mplus (Muthén & Muthén, 2012) to estimate a bifactor model of psychopathology, composed of a general factor and five specific symptom dimensions derived from the 59 psychopathology-items (Quattrone et al., 2019).

Full scale-IQ scores were calculated by WAIS-abbreviated (11/4* sum of scaled scores), based on standardised scaled scores obtained from four different subtests: Digit Symbol Substitution (Processing Speed), Arithmetic (Working Memory), Block Design (Perceptual Reasoning) and Information (Verbal Comprehension) subtests (Velthorst et al., 2013).

Principal Component Analysis reduced the nine PAS dimensions, retaining the Social Factor (PSF) (Cronbach’s Alpha=0.86) and the Academic Factor (PAF) (Cronbach’s Alpha=0.814), as described above, based on eigenvalues over the Kaiser threshold of 1, which explained 64.4% of the variance (Ferraro et al., 2019), PAS scores were reversed so that higher scores indicated better adjustment (González-Blanch et al., 2015) to allow easier comparison with IQ scores.

## **Supplementary Table 1. ICD-10 Diagnoses**

| Diagnosis according to ICD-10 from OPCRIT | Frequency | Percent |
| --- | --- | --- |
| Bipolar Affective Disorders | 104 | 13.2 |
| Depressive Disorders with Psychotic Features | 88 | 11.2 |
| Schizoaffective Disorders | 24 | 3.2 |
| Delusional disorder | 33 | 4.2 |
| Schizophrenia | 253 | 32.2 |
| Other non-organic psychotic syndrome | 283 | 36.2 |
| Total | 785 | 100 |

# *Pearson Correlations Between Symptom Dimensions, IQ, and Premorbid Characteristics*

Higher IQ was correlated with fewer positive (r=-0.12, p=0.001), negative (r=-0.15, p=0.000). disorganisation symptoms (r=-0.09, p=0.009), and more manic symptoms (r=0.09, p=0.011). Higher PSF related to fever negative (r=-0.15, p=0.000) and depressive symptoms (r=-0.14, p=0.000) and more manic symptoms (r=0.09, p=0.006). Higher PAF was correlated with higher positive (r=-0.13, p=0.000) and negative (r=-0.09, p=0.010) symptoms (Supplementary Table 2).

| **Supplementary Table 2. Pearson Correlations Between Symptom Dimensions. IQ. and Premorbid Characteristics** | | | | |
| --- | --- | --- | --- | --- |
|  | | **IQ** | **PSF** | **PAF** |
| **GENERAL** | PearsonCorrelation | -0.008 | 0.023 | 0.011 |
|  | Sig. (2-tailed) | 0.817 | 0.524 | 0.748 |
|  | N | 785 | 785 | 785 |
| **POSITIVE** | PearsonCorrelation | **-0.119^**^** | -0.053 | **-0.092^**^** |
|  | Sig. (2-tailed) | **0.001** | 0.140 | **0.010** |
|  | N | 785 | 785 | 785 |
| **NEGATIVE** | PearsonCorrelation | **-0.154^**^** | **-0.135^**^** | **-0.091^*^** |
|  | Sig. (2-tailed) | **0.000** | **0.000** | **0.010** |
|  | N | 785 | 785 | 785 |
| **DISORGANIZATION** | PearsonCorrelation | **-0.093^**^** | -0.055 | -0.010 |
|  | Sig. (2-tailed) | **0.009** | 0.125 | 0.780 |
|  | N | 785 | 785 | 785 |
| **MANIA** | PearsonCorrelation | **0.091^*^** | **0.098^**^** | 0.030 |
|  | Sig. (2-tailed) | **0.011** | **0.006** | 0.397 |
|  | N | 785 | 785 | 785 |
| **DEPRESSION** | PearsonCorrelation | 0.003 | **-0.145^**^** | 0.047 |
|  | Sig. (2-tailed) | 0.931 | **0.000** | 0.188 |
|  | N | 785 | 785 | 785 |

# *The role of cannabis use*

Lifetime cannabis use was associated with more prominent positive symptoms in our sample of FEP, as already suggested by other studies (Ringen et al., 2016; Seddon et al., 2016), and in a dose-dependent manner (Quattrone et al., 2019).

Particularly, we found here a role of processing speed interacting with cannabis use in predicting more positive symptoms.

A residual effect of cannabis (less than 28 days of abstinence) on processing speed has been observed (Jacobus et al., 2009), but no longer present three months after cessation (Fried et al., 2005). Patients in our sample who smoked cannabis daily in their lifetime are also more likely to currently smoke cannabis (Di Forti et al., 2019). Thus, we can speculate that the effect of daily cannabis use on higher positive symptoms might be related to a directly toxic influence of cannabis. Alternatively, in a sub-group of individuals with psychosis who are, in principle, less cognitively and premorbidly impaired (Ferraro et al., 2019) an overuse of cannabis might result in dopamine supersensitivity (Murray et al., 2014; Thompson et al., 2013) and predisposing them to higher positive symptoms. Subjects’ occasional users, whose pattern of cannabis use was less risky for psychosis (Di Forti et al., 2019) had, instead, the most cognitively-related positive symptoms, i.e. very low if processing speed is high. Notably, never users presented the lowest mean level of positive symptoms, regardless their processing speed abilities. This is in line with the finding of a predominance of negative symptoms in this subgroup, instead (Quattrone et al., 2020).

However, we did not find the association between lifetime cannabis use and fewer negative symptoms reported in the previous study (Quattrone et al., 2019); it was probably here absorbed by the effects of current cognitive characteristics and premorbid adjustment. This is consistent with our interpretation of previous findings, where we had proposed that cannabis use would not have a direct effect on negative symptoms. Rather, a presentation with prominent negative symptomatology would occur more likely in those individuals who never tried cannabis, as this would be an epiphenomenon of a greater neurodevelopmental impairment in psychosis. More specifically, the subgroup of patients with prominent negative symptoms at FEP would lack social skills necessary to get an illicit substance or to be part of a peer group that uses it (Quattrone et al., 2020). Hence, neurodevelopmental features may mediate the relationship between cannabis use and fewer negative symptoms, consistently with the evidence of an inverse relationship between lower IQ and the likelihood to be exposed to the substance (Ferraro et al., 2013, 2019; Murray et al., 2017; Quattrone et al., 2019).

## **Supplementary-Figure 1. Grouped Scatter Mean of Predicted Value for Positive Symptoms by Processing Speed by Frequency of cannabis Use**


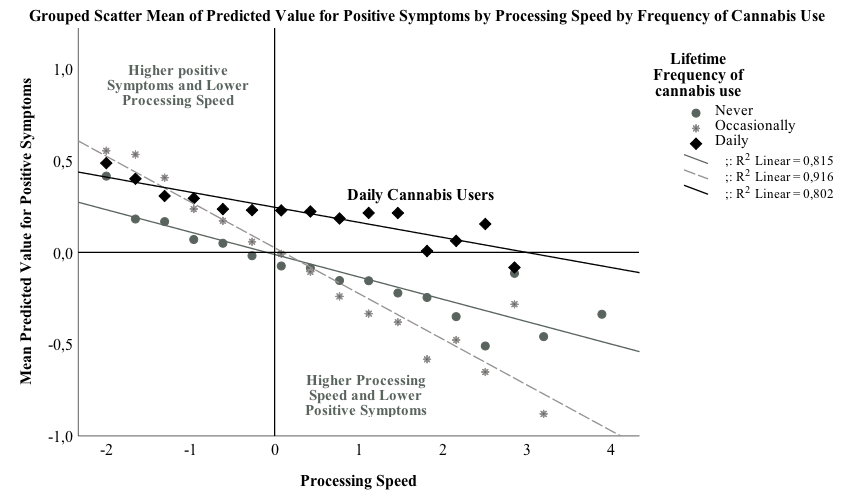


Higher Positive Symptoms and Lower Processing Speed

Lower Positive Symptoms and Higher Processing Speed

# *The effect of the other predictors*

The predictors country [Pillai=0.321, F(30, 3845)=8.8; p<0.001; partial η^2^=0.064], self-reported ethnicity [Pillai=0.029, (12, 1532)=1.8; p=0.034; partial η^2^=0.014], sex [Pillai=0.019, (6, 765)=2.5; p=0.022; partial η^2^=0.019], and age [Pillai=0.051, (6, 765)=6.9; p<0.001; partial η^2^=0.051] were all associated with symptom dimension scores.

Specifically, men presented more negative (B=0.207, 95% C.I. 0.058, 0.357, p=0.007) and disorganisation (B=0.148, 95% C.I. 0.002, 0.295, p=0.047) symptoms than women. Being younger at FEP was associated with more negative (B=-0.014, 95% C.I. -0.021, -0.007, p<0.001) and manic (B=-0.014, 95% C.I. -0.021, -0.006, p<0.001) symptoms, and fewer depressive symptoms (BB=0.012, 95% C.I. -0.005, 0.019, p=0.001). Self-reported ethnicity was not associated with any symptom dimensions.

The country [Pillai=0.321, F(30, 3845)=8.8; p<0.001; partial η2=0.064] was associated with all symptom dimension scores. That means, each country presented with a slightly specific symptom profile and showed some differences in comparisons.

Using UK patients as the reference category, they had fewer general symptoms than patients from Spain (B=-0.579, 95% C.I. -0.876, -0.281, p<0.001), but more than those from Brazil (B=0.476, 95% C.I. 0.093, 0.859, p=0.004); they had fewer negative symptoms than patients from Italy (B=-0.566, 95% C.I. -0.968, -0.164, p=0.001), and Spain (B=-0.650, 95% C.I. -1.003, -0.297, p<0.001), and fewer negative (B=-0.454, 95% C.I. -0.896, -0.011, p=0.039) and disorganised symptoms (B=-0.440, 95% C.I. -0.874, -0.006, p=0.044) than patients from France. They also manifested fewer manic symptoms than patients from the Netherlands (B=-0.417, 95% C.I. -0.746, -0.087, p=0.003). Detailed between-group comparisons are described in the Supplementary Table 3.

## **Supplementary Table 3. Parameter Estimates in the Predictive Model for Symptom Dimensions by PSF, PAF and IQ**

| **Dependent Variable** | **Parameter** | **Contrast** | **B^a^** | **SE** | ***p-value*^b^** | **95% C.I. for B ^b^** | |
| --- | --- | --- | --- | --- | --- | --- | --- |
| **GENERAL** | Gender | Males vs females | -0.027 | 0.064 | 0.671 | -0.153 | 0.099 |
|  | Age |  | -0.004 | 0.003 | 0.159 | -0.010 | 0.002 |
|  | Ethnicity | White vs black | 0.066 | 0.095 | 1.000 | -0.161 | 0.292 |
|  |  | White vs other ethnicities | 0.151 | 0.079 | 0.168 | -0.038 | 0.340 |
|  |  | Black vs other ethnicities | 0.085 | 0.104 | 1.000 | -0.163 | 0.334 |
|  | Country | UK vs Netherlands  UK vs Spain  UK vs France  UK vs Italy  UK vs Brazil | -0.155 | 0.097 | 1.000 | -0.440 | 0.131 |
|  |  |  | -0.579^*^ | 0.101 | 0.000 | -0.876 | -0.281 |
|  |  |  | 0.118 | 0.127 | 1.000 | -0.256 | 0.491 |
|  |  |  | -0.289 | 0.115 | 0.185 | -0.628 | 0.050 |
|  |  |  | 0.394^*^ | 0.101 | 0.002 | 0.097 | 0.691 |
|  |  | Netherlands vs Spain  Netherlands vs France  Netherlands vs Italy  Netherlands vs Brazil | -0.424^*^ | 0.092 | 0.000 | -0.696 | -0.152 |
|  |  |  | 0.272 | 0.124 | 0.429 | -0.093 | 0.637 |
|  |  |  | -0.134 | 0.109 | 1.000 | -0.454 | 0.186 |
|  |  |  | 0.549^*^ | 0.096 | 0.000 | 0.266 | 0.831 |
|  |  | Spain vs France  Spain vs Italy  Spain vs Brazil | 0.696^*^ | 0.124 | 0.000 | 0.330 | 1.063 |
|  |  |  | 0.290 | 0.106 | 0.098 | -0.023 | 0.603 |
|  |  |  | 0.973^*^ | 0.098 | 0.000 | 0.685 | 1.261 |
|  |  | France vs Italy  France vs Brazil | -0.406^*^ | 0.136 | 0.045 | -0.808 | -0.005 |
|  |  |  | 0.277 | 0.126 | 0.418 | -0.093 | 0.646 |
|  |  | Italy vs Brazil | 0.683^*^ | 0.110 | 0.000 | 0.358 | 1.008 |
| **POSITIVE** | Gender | Males vs females | 0.046 | 0.083 | 0.577 | -0.116 | 0.209 |
|  | Age |  | 0.006 | 0.004 | 0.130 | -0.002 | 0.014 |
|  | Ethnicity | White vs black | -0.225 | 0.122 | 0.195 | -0.518 | 0.067 |
|  |  | White vs other ethnicities | -0.240 | 0.102 | 0.056 | -0.484 | 0.004 |
|  |  | Black vs other ethnicities | -0.015 | 0.134 | 1.000 | -0.336 | 0.306 |
|  | Country | UK vs Netherlands  UK vs Spain  UK vs France  UK vs Italy  UK vs Brazil | 0.049 | 0.125 | 1.000 | -0.320 | 0.417 |
|  |  |  | 0.275 | 0.130 | 0.528 | -0.109 | 0.659 |
|  |  |  | -0.121 | 0.163 | 1.000 | -0.602 | 0.360 |
|  |  |  | 0.337 | 0.148 | 0.352 | -0.100 | 0.774 |
|  |  |  | 0.476^*^ | 0.130 | 0.004 | 0.093 | 0.859 |
|  |  | Netherlands vs Spain  Netherlands vs France  Netherlands vs Italy  Netherlands vs Brazil | 0.226 | 0.119 | 0.863 | -0.124 | 0.577 |
|  |  |  | -0.170 | 0.160 | 1.000 | -0.641 | 0.301 |
|  |  |  | 0.288 | 0.140 | 0.601 | -0.124 | 0.701 |
|  |  |  | 0.427^*^ | 0.124 | 0.009 | 0.063 | 0.791 |
|  |  | Spain vs France  Spain vs Italy  Spain vs Brazil | -0.396 | 0.161 | 0.207 | -0.869 | 0.077 |
|  |  |  | 0.062 | 0.137 | 1.000 | -0.342 | 0.465 |
|  |  |  | 0.201 | 0.126 | 1.000 | -0.171 | 0.572 |
|  |  | France vs Italy  France vs Brazil | 0.458 | 0.176 | 0.141 | -0.060 | 0.976 |
|  |  |  | 0.597^*^ | 0.162 | 0.004 | 0.120 | 1.074 |
|  |  | Italy vs Brazil | 0.139 | 0.142 | 1.000 | -0.281 | 0.558 |
| **NEGATIVE** | Gender | Males vs females | 0.207 | 0.076 | 0.007 | 0.058 | 0.357 |
|  | Age |  | -0.007 | 0.004 | 0.067 | -0.014 | 0.000 |
|  | Ethnicity | White vs black | -0.094 | 0.112 | 1.000 | -0.363 | 0.175 |
|  |  | White vs other ethnicities | -0.005 | 0.094 | 1.000 | -0.230 | 0.219 |
|  |  | Black vs other ethnicities | 0.089 | 0.123 | 1.000 | -0.206 | 0.384 |
|  | Country | UK vs Netherlands  UK vs Spain  UK vs France  UK vs Italy  UK vs Brazil | -0.312 | 0.115 | 0.103 | -0.650 | 0.027 |
|  |  |  | -0.650^*^ | 0.120 | 0.000 | -1.003 | -0.297 |
|  |  |  | -0.454^*^ | 0.150 | 0.039 | -0.896 | -0.011 |
|  |  |  | -0.566^*^ | 0.136 | 0.001 | -0.968 | -0.164 |
|  |  |  | -0.237 | 0.120 | 0.713 | -0.589 | 0.115 |
|  |  | Netherlands vs Spain  Netherlands vs France  Netherlands vs Italy  Netherlands vs Brazil | -0.339^*^ | 0.109 | 0.031 | -0.661 | -0.016 |
|  |  |  | -0.142 | 0.147 | 1.000 | -0.575 | 0.291 |
|  |  |  | -0.254 | 0.129 | 0.732 | -0.633 | 0.125 |
|  |  |  | 0.074 | 0.114 | 1.000 | -0.260 | 0.409 |
|  |  | Spain vs France  Spain vs Italy  Spain vs Brazil | 0.197 | 0.148 | 1.000 | -0.238 | 0.631 |
|  |  |  | 0.084 | 0.126 | 1.000 | -0.287 | 0.455 |
|  |  |  | 0.413^*^ | 0.116 | 0.006 | 0.071 | 0.754 |
|  |  | France vs Italy  France vs Brazil | -0.112 | 0.162 | 1.000 | -0.588 | 0.364 |
|  |  |  | 0.216 | 0.149 | 1.000 | -0.222 | 0.655 |
|  |  | Italy vs Brazil | 0.329 | 0.131 | 0.184 | -0.057 | 0.714 |
| **DISORGANIZATION** | Gender | Males vs females | 0.148 | 0.075 | 0.047 | 0.002 | 0.295 |
|  | Age |  | -0.014 | 0.004 | 0.000 | -0.021 | -0.007 |
|  | Ethnicity | White vs black | -0.256 | 0.110 | 0.061 | -0.519 | 0.008 |
|  |  | White vs other ethnicities | -0.209 | 0.092 | 0.069 | -0.429 | 0.011 |
|  |  | Black vs other ethnicities | 0.047 | 0.121 | 1.000 | -0.243 | 0.336 |
|  | Country | UK vs Netherlands  UK vs Spain  UK vs France  UK vs Italy  UK vs Brazil | -0.293 | 0.113 | 0.143 | -0.625 | 0.039 |
|  |  |  | -0.169 | 0.118 | 1.000 | -0.516 | 0.177 |
|  |  |  | -0.440^*^ | 0.147 | 0.044 | -0.874 | -0.006 |
|  |  |  | -0.125 | 0.134 | 1.000 | -0.519 | 0.269 |
|  |  |  | 0.127 | 0.117 | 1.000 | -0.219 | 0.472 |
|  |  | Netherlands vs Spain  Netherlands vs France  Netherlands vs Italy  Netherlands vs Brazil | 0.124 | 0.107 | 1.000 | -0.192 | 0.440 |
|  |  |  | -0.147 | 0.144 | 1.000 | -0.571 | 0.278 |
|  |  |  | 0.168 | 0.126 | 1.000 | -0.204 | 0.540 |
|  |  |  | 0.420^*^ | 0.112 | 0.003 | 0.091 | 0.748 |
|  |  | Spain vs France  Spain vs Italy  Spain vs Brazil | -0.270 | 0.145 | 0.933 | -0.697 | 0.156 |
|  |  |  | 0.045 | 0.124 | 1.000 | -0.319 | 0.408 |
|  |  |  | 0.296 | 0.114 | 0.143 | -0.039 | 0.631 |
|  |  | France vs Italy  France vs Brazil | 0.315 | 0.159 | 0.711 | -0.152 | 0.782 |
|  |  |  | 0.566^*^ | 0.146 | 0.002 | 0.136 | 0.996 |
|  |  | Italy vs Brazil | 0.251 | 0.128 | 0.762 | -0.127 | 0.629 |
| **MANIA** | Gender | Males vs females | -0.128 | 0.077 | 0.098 | -0.279 | 0.024 |
|  | Age |  | -0.014 | 0.004 | 0.000 | -0.021 | -0.006 |
|  | Ethnicity | White vs black | 0.016 | 0.114 | 1.000 | -0.256 | 0.289 |
|  |  | White vs other ethnicities | -0.065 | 0.095 | 1.000 | -0.292 | 0.163 |
|  |  | Black vs other ethnicities | -0.081 | 0.125 | 1.000 | -0.380 | 0.218 |
|  | Country | UK vs Netherlands  UK vs Spain  UK vs France  UK vs Italy  UK vs Brazil | -0.171 | 0.117 | 1.000 | -0.515 | 0.172 |
|  |  |  | 0.343 | 0.122 | 0.073 | -0.015 | 0.701 |
|  |  |  | -0.035 | 0.152 | 1.000 | -0.484 | 0.413 |
|  |  |  | 0.124 | 0.138 | 1.000 | -0.283 | 0.532 |
|  |  |  | 0.076 | 0.121 | 1.000 | -0.281 | 0.433 |
|  |  | Netherlands vs Spain  Netherlands vs France  Netherlands vs Italy  Netherlands vs Brazil | 0.514^*^ | 0.111 | 0.000 | 0.188 | 0.841 |
|  |  |  | 0.136 | 0.149 | 1.000 | -0.303 | 0.575 |
|  |  |  | 0.296 | 0.131 | 0.357 | -0.089 | 0.680 |
|  |  |  | 0.247 | 0.115 | 0.482 | -0.092 | 0.587 |
|  |  | Spain vs France  Spain vs Italy  Spain vs Brazil | -0.378 | 0.150 | 0.175 | -0.819 | 0.062 |
|  |  |  | -0.219 | 0.128 | 1.000 | -0.595 | 0.157 |
|  |  |  | -0.267 | 0.118 | 0.352 | -0.613 | 0.079 |
|  |  | France vs Italy  France vs Brazil | 0.160 | 0.164 | 1.000 | -0.323 | 0.642 |
|  |  |  | 0.111 | 0.151 | 1.000 | -0.333 | 0.556 |
|  |  | Italy vs Brazil | -0.048 | 0.133 | 1.000 | -0.439 | 0.343 |
| **DEPRESSION** | Gender | Males vs females | -0.066 | 0.074 | 0.371 | -0.212 | 0.079 |
|  | Age |  | 0.012 | 0.004 | 0.001 | 0.005 | 0.019 |
|  | Ethnicity | White vs black | 0.090 | 0.109 | 1.000 | -0.172 | 0.352 |
|  |  | White vs other ethnicities | 0.068 | 0.091 | 1.000 | -0.151 | 0.286 |
|  |  | Black vs other ethnicities | -0.022 | 0.120 | 1.000 | -0.310 | 0.265 |
|  | Country | UK vs Netherlands  UK vs Spain  UK vs France  UK vs Italy  UK vs Brazil | -0.417^*^ | 0.112 | 0.003 | -0.746 | -0.087 |
|  |  |  | 0.123 | 0.117 | 1.000 | -0.221 | 0.466 |
|  |  |  | -0.026 | 0.146 | 1.000 | -0.457 | 0.405 |
|  |  |  | -0.224 | 0.133 | 1.000 | -0.615 | 0.167 |
|  |  |  | 0.156 | 0.116 | 1.000 | -0.186 | 0.499 |
|  |  | Netherlands vs Spain  Netherlands vs France  Netherlands vs Italy  Netherlands vs Brazil | 0.539^*^ | 0.107 | 0.000 | 0.226 | 0.853 |
|  |  |  | 0.391 | 0.143 | 0.097 | -0.031 | 0.812 |
|  |  |  | 0.193 | 0.125 | 1.000 | -0.176 | 0.562 |
|  |  |  | 0.573^*^ | 0.111 | 0.000 | 0.247 | 0.899 |
|  |  | Spain vs France  Spain vs Italy  Spain vs Brazil | -0.149 | 0.144 | 1.000 | -0.572 | 0.274 |
|  |  |  | -0.346 | 0.123 | 0.073 | -0.707 | 0.015 |
|  |  |  | 0.034 | 0.113 | 1.000 | -0.299 | 0.366 |
|  |  | France vs Italy  France vs Brazil | -0.198 | 0.157 | 1.000 | -0.661 | 0.266 |
|  |  |  | 0.182 | 0.145 | 1.000 | -0.244 | 0.609 |
|  |  | Italy vs Brazil | 0.380^*^ | 0.127 | 0.044 | 0.005 | 0.755 |

Based on estimated marginal means

^a^ Mean difference

^b^ Adjustment for multiple comparisons: Bonferroni.

The differences found in symptom dimensions by age, gender, ethnicity and countries were expected, based on our previous study. About country we found, for example, that positive symptoms are more present in patients from minority ethnic groups, as compared with majority populations, who are mostly represented in some countries (e.g. France or UK,). Additionally, we found that residents in urban areas scored higher on the general symptom dimension, as compared to rural areas residents (e.g. some sites in Italy and Spain) (Quattrone et al., 2019).

# *DUP and AP treatment influence on the relationship between symptom dimensions and cognitive and premorbid adjustment*

Patients had a median DUP of 61.5 week (sd=179). 61.8% (N=485) of patients were AP free at the time of the assessment, 22% (N=173) was taking 1 AP, 16.2% (N=127) was taking more than 1 AP. A longer DUP was related to lower PSA (r=-0.124, p<0.001), lower general (r=-0.124, p<0.001) and higher positive symptoms (r=0.170, p<0.001). Patients with more than one AP had more negative and disorganised symptoms than patients with one AP (Mean difference for negative = 0.354; C.I. 95% 0.073, 0.635, p=0.008; Mean difference for disorganisation = 0.277; C.I. 95% 0.002, 0.551, p=0.047) or AP free (Mean difference for negative=0.271; C.I. 95% 0.032, 0.511, p=0.020; Mean difference for disorganisation = 0.306; C.I. 95% 0.072, 0. 540, p=0.005) at the time of the interview. We wanted to repeat the MANCOVA by including DUP and AP treatment as covariates, to avoid any influence of long-lasting symptomatology and pharmacotherapy on the relationship between the variables of interest. AP had no effect into the model [Pillai=0.020, F(12, 1406)=1.17; p=0.300; partial η2=0.010], DUP was instead significantly related to the results [Pillai=0.042, F(6, 702)=5.121; p<0.001; partial η2=0.042]. This measure was related to the same symptom dimensions as in the bivariate correlations, but it did not influence the results. In fact, positive symptoms were still more common in individuals with lower IQ (B=-0.006, 95% C.I. -0.011, -0.001, p=0.024). Negative symptoms were associated with lower IQ (B=-0.005, 95% C.I. -0.010, 0.0, p=0.038), and with worse PSF (B=-0.139, 95% C.I. -0.20, -0.07, p<0.001). Manic symptoms were still more common in patients with higher IQ (B=0.006, 95% C.I. 0.001, 0.010, p=0.017), and better PSF (B=0.076, 95% C.I. 0.144, 0.007, p=0.008). Depressive symptoms were more common in individuals with lower PSF (B=-0.091, 95% C.I. -0.156, -0.027, p=0.006). This analysis, did not reveal any association between IQ and/or PSF with the general psychosis factor or the disorganisation symptom dimensions and no novel associations were found.

# **References**

Di Forti, M., Quattrone, D., Freeman, T. P., Tripoli, G., Gayer-Anderson, C., Quigley, H., Rodriguez, V., Jongsma, H. E., Ferraro, L., La Cascia, C., La Barbera, D., Tarricone, I., Berardi, D., Szöke, A., Arango, C., Tortelli, A., Velthorst, E., Bernardo, M., Del-Ben, C. M., … van der Ven, E. (2019). The contribution of cannabis use to variation in the incidence of psychotic disorder across Europe (EU-GEI): a multicentre case-control study. *The Lancet Psychiatry*, *6*(5), 427–436. https://doi.org/10.1016/S2215-0366(19)30048-3

Ferraro, L., Cascia, C. La, Quattrone, D., Sideli, L., Matranga, D., Capuccio, V., Tripoli, G., Gayer-Anderson, C., Morgan, C., Sami, M. B., Sham, P., de Haan, L., Velthorst, E., Jongsma, H. E., Kirkbride, J. B., Rutten, B. P. F., Richards, A. L., Roldan, L., Arango, C., … Forti, M. Di. (2019). Premorbid Adjustment and {IQ} in Patients With First-Episode Psychosis: A Multisite Case-Control Study of Their Relationship With Cannabis Use. *Schizophrenia Bulletin*. https://doi.org/10.1093/schbul/sbz077

Ferraro, L., Russo, M., O’Connor, J., Wiffen, B. D. R., Falcone, M. A., Sideli, L., Gardner-Sood, P., Stilo, S., Trotta, A., Dazzan, P., Mondelli, V., Taylor, H., Friedman, B., Sallis, H., La Cascia, C., La Barbera, D., David, A. S., Reichenberg, A., Murray, R. M., & Di Forti, M. (2013). Cannabis users have higher premorbid IQ than other patients with first onset psychosis. *Schizophrenia Research*, *150*(1), 129–135.

Fried, P., Watkinson, B., & Gray, R. (2005). Neurocognitive consequences of marihuana-a comparison with pre-drug performance. *Neurotoxicology and Teratology*, *27*(2), 231–239.

González-Blanch, C., Gleeson, J. F., Koval, P., Cotton, S. M., McGorry, P. D., & Alvarez-Jimenez, M. (2015). Social functioning trajectories of young first-episode psychosis patients with and without cannabis misuse:A 30-month follow-up study. *PLoS ONE*, *10*(4), e0122404. https://doi.org/10.1371/journal.pone.0122404

Jacobus, J., Bava, S., Cohen-Zion, M., Mahmood, O., & Tapert, S. F. (2009). Functional consequences of marijuana use in adolescents. *Pharmacology, Biochemistry, and Behavior*, *92*(4), 559–565.

Murray, R. M., Englund, A., Abi-Dargham, A., Lewis, D. A., Di Forti, M., Davies, C., Sherif, M., McGuire, P., & D’Souza, D. C. (2017). Cannabis-associated psychosis: Neural substrate and clinical impact. In *Neuropharmacology* (Vol. 124, pp. 89–104). Pergamon. https://doi.org/10.1016/j.neuropharm.2017.06.018

Murray, R. M., Mehta, M., & Di Forti, M. (2014). Different Dopaminergic abnormalities underlie cannabis dependence and cannabis-induced psychosis. In *Biological Psychiatry* (Vol. 75, Issue 6, pp. 430–431). https://doi.org/10.1016/j.biopsych.2014.01.011

Muthén, L. K., & Muthén, B. O. (2012). *Mplus : statistical analysis with latent variables : user’s guide*. www.StatModel.com

Quattrone, D., Di Forti, M., Gayer-Anderson, C., Ferraro, L., Jongsma, H. E., Tripoli, G., La Cascia, C., La Barbera, D., Tarricone, I., Berardi, D., Szöke, A., Arango, C., Lasalvia, A., Tortelli, A., Llorca, P. M., De Haan, L., Velthorst, E., Bobes, J., Bernardo, M., … Cristofalo, D. (2019). Transdiagnostic dimensions of psychopathology at first episode psychosis: Findings from the multinational EU-GEI study. *Psychological Medicine*, *49*(8), 1378–1391. https://doi.org/10.1017/S0033291718002131

Quattrone, D., Ferraro, L., Tripoli, G., La Cascia, C., Quigley, H., Quattrone, A., Jongsma, H., Del Peschio, S., Gatto, G., EU-GEI_GROUP, Gayer-Anderson, C., Jones, P. B., Kirkbride, J. B., La Barbera, D., Tarricone, I., Tosato, S., Lasalvia, A., Szöke, A., Arango, C., … Di Forti, M. (2020). Daily use of high potency cannabis is associated with more positive symptoms in first episode psychosis patients: the EU-GEI case-control study. *Psychological Medicine*, *in press*.

Ringen, P. A., Nesvåg, R., Helle, S., Lagerberg, T. V., Lange, E. H., Loberg, E. M., Agartz, I., Andreassen, O. A., & Melle, I. (2016). Premorbid cannabis use is associated with more symptoms and poorer functioning in schizophrenia spectrum disorder. *Psychological Medicine*, *46*(15), 3127–3136. https://doi.org/10.1017/S0033291716001999

Seddon, J. L., Birchwood, M., Copello, A., Everard, L., Jones, P. B., Fowler, D., Amos, T., Freemantle, N., Sharma, V., Marshall, M., & Singh, S. P. (2016). Cannabis Use Is Associated With Increased Psychotic Symptoms and Poorer Psychosocial Functioning in First-Episode Psychosis: A Report From the UK National EDEN Study. *Schizophrenia Bulletin*, *42*(3), 619–625. https://doi.org/10.1093/schbul/sbv154

Thompson, J. L., Urban, N., Slifstein, M., Xu, X., Kegeles, L. S., Girgis, R. R., Beckerman, Y., Harkavy-Friedman, J. M., Gil, R., & Abi-Dargham, A. (2013). Striatal dopamine release in schizophrenia comorbid with substance dependence. *Molecular Psychiatry*, *18*(8), 909–915. https://doi.org/10.1038/mp.2012.109

Velthorst, E., Levine, S. Z., Henquet, C., De Haan, L., Van Os, J., Myin-Germeys, I., & Reichenberg, A. (2013). To cut a short test even shorter: Reliability and validity of a brief assessment of intellectual ability in Schizophrenia - A control-case family study. *Cognitive Neuropsychiatry*, *18*(6), 574–593. https://doi.org/10.1080/13546805.2012.731390
